# Electrochemical Biosensors for Cytokine Profiling: Recent Advancements and Possibilities in the Near Future

**DOI:** 10.3390/bios11030094

**Published:** 2021-03-23

**Authors:** Nirmita Dutta, Peter B. Lillehoj, Pedro Estrela, Gorachand Dutta

**Affiliations:** 1 School of Medical Science and Technology (SMST), Indian Institute of Technology Kharagpur, Kharagpur 721302, India; nirmita_dutta@iitkgp.ac.in; 2 Department of Mechanical Engineering, Rice University, Houston, TX 77005, USA; lillehoj@rice.edu; 3 Centre for Biosensors, Bioelectronics and Biodevices (C3Bio) and Department of Electronic & Electrical Engineering, University of Bath, Bath BA2 7AY, UK

**Keywords:** cytokine, electrochemical, biosensor, cancer, autoimmunity, neurodegeneration

## Abstract

Cytokines are soluble proteins secreted by immune cells that act as molecular messengers relaying instructions and mediating various functions performed by the cellular counterparts of the immune system, by means of a synchronized cascade of signaling pathways. Aberrant expression of cytokines can be indicative of anomalous behavior of the immunoregulatory system, as seen in various illnesses and conditions, such as cancer, autoimmunity, neurodegeneration and other physiological disorders. Cancer and autoimmune diseases are particularly adept at developing mechanisms to escape and modulate the immune system checkpoints, reflected by an altered cytokine profile. Cytokine profiling can provide valuable information for diagnosing such diseases and monitoring their progression, as well as assessing the efficacy of immunotherapeutic regiments. Toward this goal, there has been immense interest in the development of ultrasensitive quantitative detection techniques for cytokines, which involves technologies from various scientific disciplines, such as immunology, electrochemistry, photometry, nanotechnology and electronics. This review focusses on one aspect of this collective effort: electrochemical biosensors. Among the various types of biosensors available, electrochemical biosensors are one of the most reliable, user-friendly, easy to manufacture, cost-effective and versatile technologies that can yield results within a short period of time, making it extremely promising for routine clinical testing.

## 1. Introduction to Cytokines

Cytokines are a class of soluble signaling proteins, ranging in size from 8 to 40 kDa, which act as mediators of innate and adaptive immunity. Cytokines are secreted in response to an inflammatory stimulus by nearly all nucleated cells, particularly immune cells or leucocytes. While structurally versatile, cytokines are grouped together based on their biological functions, which are similar in principle and often orchestrated in an interdependent manner [1,2].

Various types of inflammatory stimulus, such as antigenic stimulation, UV light, heat-shock or other forms of stress inducer, can result in the production of cytokines [1]. Antigenic pathogen associated molecular patterns (PAMPS) and damage associated molecular patterns (DAMPS) trigger an immune response when encountered by immune cells at the site of an injury or pathogen invasion. PAMPS are recognized by pathogen recognition receptors (PRRs), the most important of which are Toll-like receptors (TLRs) on macrophages and dendritic cells. The result is a cascade of molecular signals transduced through common pathways, such as nuclear factor kappa-light-chain-enhancer of activated B cells (NF-κB) and mitogen-activated protein kinase (MAPK) pathways, which activate transcription factors responsible for switching on cytokine encoding genes. Major cytokine-producing sources are T cells, B cells, macrophages, dendritic cells and natural killer cells. Gene expression shows transient behavior, and the expressed cytokines, upon secretion, bind to their respective receptors on the surface of target cells, leading to further signal transduction via common pathways, such as Janus kinase-signal transducer and activator of transcription (JAK-STAT), NF-κB, MAPK and apoptotic Caspase pathways [2].

**Figure 1:**
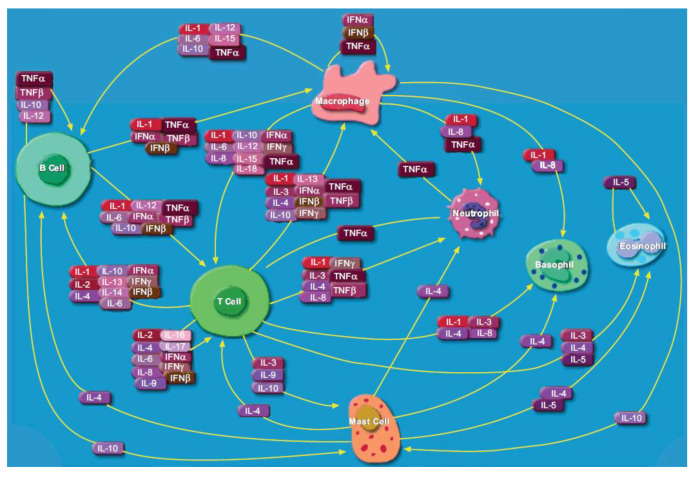
Metabolic pathways of cytokines in humans. Reprinted from [4] with permission from PubMed Central.

The cytokine family includes interleukins (ILs), chemokines (CXCLs), interferons (IFNs), tumor necrosis factors (TNFs), mesenchymal growth factors, transforming growth factors (TGFs), adipocytokines and the hematopoietic growth factors [2,3]. Cytokines do not function independently, but act in sync with each other in a tight-knit immunoregulatory system (shown in Figure 1 [4]). Cytokine function is accomplished by multiple mechanisms, including pleiotropy (cytokines conveying different signals in different target cells), redundancy (multiple cytokines conveying similar signals), synergism (multiple cytokines conveying a signal combinatorially), antagonism (cytokines conveying opposing or inhibitory signals) or cascade induction (a network of cytokines successively inducing the production of more types of cytokines) [2,5]. Cytokines facilitate crosstalk between immune cells at all stages of an inflammatory response, which can either promote (pro-inflammatory) and or reduce (anti-inflammatory) this response [1,6]. Cytokines also work in close association with antigen recognition molecules, such as T cell receptors, B cell receptors, major histocompatibility complex classes I and II (MHC I and II) and PRRs, on the surface of antigen presenting cells. Some cytokines can induce their own production and amplify the target immune response, while others can regulate the class switching of antibody encoding genes in differentiating B cells [5]. In addition to playing an indispensable role in inflammation, cytokines play an important role in hematopoietic differentiation, embryonic development and degenerative processes [7].

### 1.1. Cytokine Expression in Infection, Cancer, Autoimmunity and Neurodegeneration

Cytokines show aberrant upregulation, or downregulation, as part of an immune response due to a disease or physiological condition, including a bacterial/viral infection, cancer, autoimmunity or neurodegeneration. In pathogenic infections, the differential expression of cytokines can provide some information about the type and extent of an acute immune response. The cytokine expression profile can also help distinguish between the immune response patterns in bacterial and viral infections [8,9]. In this respect, it is important to emphasize the potential role of cytokines in the assessment of the pathogenesis of emerging infectious diseases, such as the novel coronavirus disease (COVID-19), which is one of the deadliest pandemics in the 21st century. Recently, Dhar et al. conducted a meta-analysis to understand the correlation between the cytokines overexpressed in COVID-19 patients and disease pathogenesis. The results suggested that two cytokines, IL-6 and IL-10, showed significantly raised levels and could be quantified to estimate the risk and severity of the disease [10].

While all physiological conditions generate an immune response, cancers and autoimmune diseases are two illnesses that systematically manipulate the immune system to support their own survival [11,12]. In cancer, an inadequate or altered tumor-suppressive cytokine expression renders the immune system debilitated of its killing competence. In a study by Ashizawa et al., high serum levels of IL-6 was associated with gastric cancer and lymph node metastasis [13,14]. Yun et al. showed the similarity between the STAT phosphorylation pattern induced by the regulatory T cell-subset (T_reg_) specific cytokine milieu between colorectal cancer tumor-microenvironment and peripheral blood [15]. A study performed on human lung cancer cell lines and biopsies showed an overexpression of IL-6 and IL-10 mRNAs typical of the helper T cell-subset 2 (T_H_2) type response, overexpression of immunosuppressive TGFβ mRNA, and high secretion levels of IFNγ, granulocyte-macrophage colony-stimulating factor (GM-CSF) with reduced levels of IL-2, typical of the helper T cell-subset 1 (T_H_1) type response [16]. Another study on human bladder cancer cells established a link between increased secretion of macrophage migration inhibitory factor and cell proliferation and survival [17]. Pancreatic carcinoma cells were shown to overexpress mRNAs of various cytokines (IL-1β, IL-6, IL-10, IFNγ, TGF-β1, etc.) and IL-1β, TGF-β2, TGF-β3 proteins [18]. It has also been shown that cytokines can significantly change the tumor microenvironment by recruiting immune cells and increasing the infiltration of cytotoxic and phagocytic cells; thus, it has been proposed to use cytokines as therapeutic targets for their tumor-suppressive activity, including IFNγ, IL-2, IL-10, TGFβ and GM-CSF [19]. 

In autoimmunity, the immune system is triggered by self-antigens, which results in a systematic reaction that attacks healthy tissue. It is often mediated by autoreactive T cells which escape common checkpoint mechanisms (e.g., thymic deletion, development into natural T_reg_ or peripheral anergy), leading to an imbalance between immunostimulatory and immunosuppressive cytokines. A study by Ioannou and Isenberg demonstrated an association between high levels of IL-2 secreted by T_H_ cells with thyroiditis, rheumatoid arthritis and other arthropathies [20]. IL-12 subunit p40 encoding gene polymorphism has been associated with type I diabetes in humans [21]. Autoimmune diseases are found to be inhibited by the increased secretion of anti-inflammatory IL-10 and TGFβ by T_reg_ cells [22]. Neurodegeneration is the result of rapid damage and eventual death of neurons in a delayed response to injury. Inflammatory pathways might have a role to play here, though infiltration by immune cells in the central nervous system is barred, but inducible. There has been evidence of the involvement of IL-1, TNFα (promoting) and TGFβ (protective) in neurodegenerative processes [23]. The manipulation of cytokine expression and serum concentration can alter the direction and severity of an immune response, and serve as a potential target for immunotherapeutic procedures [24]. 

### 1.2. Important Cytokines as Potential Biomarkers

(i) Interleukin-6 (IL-6) is a 21–26 kDa glycoprotein, 212 amino acid residues long, with a pleiotropic effect, secreted in higher levels in response to an inflammatory stimulus [25,26]. It can mediate both pro- and anti-inflammatory responses by binding to its specific receptor IL-6R on target cells [27]. Secreted by leucocytes, osteoblasts, fibroblasts, mesenchymal, endothelial and many other cells [26], IL-6 is found in the serum of a healthy human in concentrations of ~2.91 ± 6.45 pg/mL [28]. IL-6 induces naïve B cells to proliferate and differentiate into plasma cells, induces the proliferation of T cells and pluripotent hematopoietic cells, mediates acute phase response in the liver and stimulates the production of C-reactive protein and fibrinogen [26,27]. Altered levels of IL-6 have been shown to be associated with various diseases and conditions, such as Alzheimer’s disease [29], major depressive disorders [30], cardiovascular diseases [31], cancers such as breast, prostate and gastric cancers [32], and autoimmune diseases, such as rheumatoid arthritis, chronic inflammatory proliferative disease and systemic lupus erythematosus [33].

(ii) Tumor necrosis factor alpha (TNFα) is a 157 amino acid residues long [34], ~17 kDa polypeptide that exists as a serum soluble homotrimer [35]. Primarily produced by activated macrophages, TNFα binds to its receptor TNFαR on the surface of target cells, promoting the activation of proinflammatory genes, differentiation of immune cells into effectors and even the induction of apoptotic pathways in some cells [35]. The serum level of TNFα in healthy individuals is ~3.21 ± 4.04 pg/mL [28], and altered levels of TNFα have been linked with several diseases and conditions, including autoimmune diseases such as chronic inflammatory bowel diseases (Crohn’s disease) [36], rheumatoid arthritis [20,37], ankylosing spondylitis [38] and psoriasis [39], and cancers [40]. 

(iii) Interferon gamma (IFNγ) is a ~20 kDa, 146 amino acid residues long glycosylated protein of the type II interferon family, which exists as a homodimer in blood [41,42]. Primarily secreted by NK cells and activated T cells, IFNγ binds to the IFNγ R_1_ and R_2_ receptor complex, and can inhibit viral replication, activate macrophages as part of the innate immunity, and is a major modulator of the T cell mediated immune response [42,43]. The serum level of IFNγ in healthy individuals is ~13.1 ± 22.7 pg/mL [28]. Elevated levels of IFNγ is associated with several illnesses, such as rheumatoid arthritis and multiple sclerosis [44,45], and is widely considered a clinical biomarker for early-stage tuberculosis diagnosis [46].

(iv) Transforming growth factor-beta 1 (TGF-β1) is a ~25 kDa protein [47] involved in regulatory or homeostatic function upon effector T cells and T_reg_ cells [48]. The physiological level of TGF-β1 in healthy individuals is 4.1 ±2.0 ng/mL [49]. Secretion of TGF-β1 in blood serum at elevated levels is linked to several conditions, including liver fibrosis [50], bladder carcinoma [51] and renal disease [52]. TGF-β1 is also thought to alleviate inflammation in chronic neurodegeneration [53].

### 1.3. Techniques for Cytokine Detection and Quantification

Levels of cytokines in healthy and diseased individuals generally range from pg/mL to ng/mL in blood/serum, and can be at much lower concentrations (fg/mL) in other bodily fluids, such as saliva, sweat and urine [28,54], and therefore requires highly sensitive detection techniques. Conventional methods for cytokine detection and quantification include enzyme-linked immunosorbent assay (ELISA) [55,56,57,58,59,60,61], Western Blot [56,62], flow cytometer assisted bead-based immunoassays [63,64], enzyme-linked immuno spot assay (ELISPOT) and fluorophore-linked immuno spot assay (FluoroSpot) [65]. ELISA is currently the gold standard for quantitative detection of cytokines used in clinical laboratories [66]. Other detection techniques, including those still in the process of development, include polymerase chain reaction (PCR)-based amplification of cytokine mRNAs [67], capillary electrophoresis [68], surface plasmon resonance-based assays [69], electrochemiluminescent assays [70,71,72], fluoroimmunoassays [73,74], radioimmunoassays [58,75], immune-PCR assay [76] and electrochemical assays.

Several other review articles have recently been published focusing on various aspects of cytokine detection. Stenken et al. reviewed common analytical techniques for cytokine detection [65], and Liu et al. and Mobed et al. discussed strategies for ultrasensitive detection of cytokines using immunosensors [77,78]. Loo et al. discussed the prospect of using paper-based electrochemical biosensors for cytokine and cancer biomarkers detection [13]. Recently, Campuzano et al. reviewed electrochemical biosensors, with the exception of FET-biosensors, for the detection of inflammatory cytokines published in the past five years [79]. Here, we present a comprehensive review on electrochemical biosensors for quantitative detection of cytokines for the diagnosis and assessment of human diseases and conditions, including cancers, autoimmune diseases and neurodegenerative diseases, published within the past two decades.

## 2. Brief Overview of Electrochemical Detection

**Figure 2:**
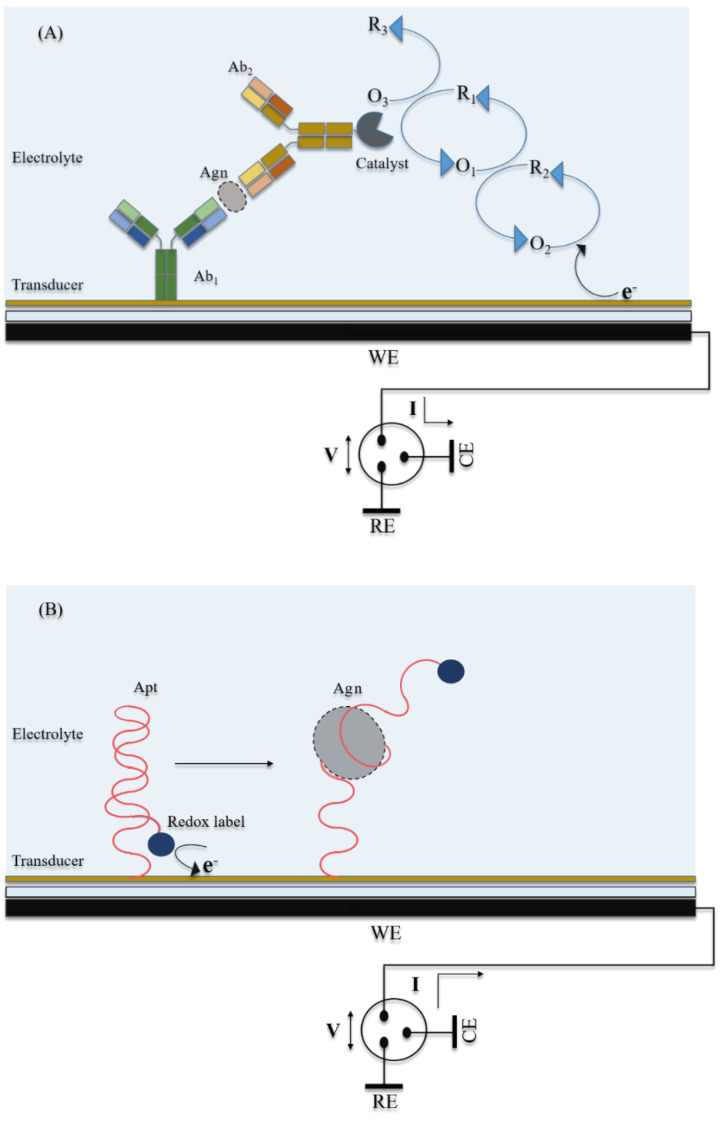
Schematic illustrations of (**A**) electrochemical immunosensor and (**B**) electrochemical aptasensor. (Agn = Antigen, Ab_1_ = Primary antibody, Ab_2_ = Secondary antibody, Apt = Aptamer, R = Reduced species, O = Oxidized species, V = Applied voltage, I = Current signal).

Electrochemical detection harnesses chemical/biochemical reactions taking place at or near an electrode surface, and the changes in the electrochemical double layer or in electron transfer characteristics at the electrode interface. The biochemical reactions that are most widely exploited are enzymatic reactions, highly specific antigen–antibody binding, and antigen-specific aptamer folding interactions. The information regarding the quantifiable biochemical reaction is relayed in the form of an electrical signal, and read in measurable electrical parameters, such as current (I), voltage (V), resistance (R), conductance (G), capacitance (C) or phase shift (θ). A simplified schematic of a typical electrochemical biosensor is shown in Figure 2.

The most common setup used to construct an electrochemical biosensor is the three-electrode system comprising a working electrode (WE, where the biochemical reactions take place to generate a proportionate current); counter electrode (CE, which completes the current loop); and a reference electrode (RE, electrically isolated, with a constant potential, against which the potential of WE is calibrated). All three electrodes are immersed in and connected by an electrolyte solution. For some electrochemical techniques, the electrolyte solution contains one or more electroactive redox species to enhance the electron transfer rate between the electrochemical reaction and sensor surface for signal amplification.

Several detection techniques are applied to electrochemical biosensors that differ in the mode of application of potential and signal generation, which can be broadly classified as potential step or potential sweep methods. Potential step methods, such as chronoamperometry (CA), involve stepping the potential applied to the WE from a base potential. The resulting current signal is correlated with the analyte concentration. Alternatively, potential sweep methods involve the gradual scanning of the WE potential from a base value, driving the redox reaction either unidirectionally or bidirectionally. Some common methods are cyclic voltammetry (CV), differential pulse voltammetry (DPV), square wave voltammetry (SWV) and anodic stripping voltammetry (ASV). CV makes use of linear scanning of the WE potential in a triangular waveform (cyclic scanning) at a constant rate. DPV uses voltage pulses of fixed amplitude superimposed on a linear potential ramp; while in SWV, the square waveform voltage of a fixed, but large, amplitude is superimposed on the potential ramp, such as to induce reversible redox reaction in each pulse. In ASV, a metal serves as the redox species, which is first deposited at the cathode at a negative potential, followed by scanning the potential in a pulse waveform-positive sweep. In the latter step, reduced metals are reoxidized and stripped out of the WE. In each of these techniques, the peak current across the potential sweep is correlated to the analyte concentration. Electrochemical impedance spectroscopy (EIS) is another technique that involves the application of an alternating, sinusoidal voltage over a range of frequencies to the WE, and the resulting electrical impedance or phase shift is correlated to the analyte concentration [80]. Apart from these, other electrochemical techniques include potentiometric approaches based on field-effect transistors (FETs). FET biosensors utilize a semiconductor-based transducer as a current path across source and drain electrodes, to which an external voltage is applied capacitatively through a gate electrode [81,82]. The authors of this review have utilized the above-mentioned electrochemical techniques to achieve ultrasensitive detection of analytes in many biosensors [81,83,84,85,86,87,88,89,90,91,92].

### 2.1. Electrochemical Biosensors for Cytokine Detection 

With the increasing discovery of new clinically relevant cytokines, and the multifunctional nature of their action, there is a pressing need for new technologies that can rapidly detect and quantify cytokines with high sensitivity and accuracy. Recent advances in nanotechnology, such as the discovery of metallic and magnetic nanoparticles, carbon nanotubes, and quantum dots [93], has enabled ultrasensitive electrochemical detection of cytokines. Various electrochemical biosensors have been demonstrated for cytokine detection which can be categorized by their sensing strategy or transduction mechanism, such as aptasensors [94], enzyme-labeled immunosensors [95], self-assembled monolayer (SAM)-enabled impedimetric immunosensors [96], heavy metal nanoparticle-labeled immunosensors [97], redox-labeled immunosensors [98], magnetoimmunosensors [99] and FET-based biosensors [100]. Additionally, biosensors exhibiting an overlap of sensing strategies or combined with other technologies (e.g., microfluidics) have been demonstrated for cytokine detection [101]. For each category of electrochemical biosensors, the design and sensor preparation process as well as sensor performance parameters are presented in Table 1, Table 2, Table 3, Table 4, Table 5, Table 6, Table 7, Table 8, Table 9, Table 10, Table 11, Table 12, Table 13, Table 14, Table 15, Table 16, Table 17 and Table 18. Among all sensor performance parameters, the most common parameters used to evaluate the sensor performance are the limit of detection (LOD), detection range, specificity, reproducibility and stability. The LOD is the lowest concentration that can be detected and is typically calculated as three times the standard deviation (SD) above the mean detection signal at zero concentration of the target biomarker [102]. A different approach to estimating the LOD is by calculating three times the SD of the blank signal, divided by the slope of the calibration plot. As different methods of calculating LOD exist, care should be taken when comparison LODs from different references. The detection range is the span of concentrations that can be detected and is often expressed as a linear regression equation. Specificity provides an indication of how accurate the biosensor can detect the target biomarker in the presence of other biomarkers and interfering species in the sample. Reproducibility provides an indication of the biosensor accuracy over multiple measurements (commonly using different sensing electrodes) and is often expressed in terms of statistical parameters, such as the SD, relative standard deviation (RSD) or coefficient of variation (CV). Most biosensors are tested using buffer samples or biofluids (blood, serum, saliva, etc.) obtained from healthy volunteers spiked with cytokines, which can provide a suitable assessment of device functionality. Further evaluation of biosensor performance is performed using clinical samples, and the results are typically compared with a gold standard technique.

#### 2.1.1. Aptasensors

**Figure 3:**
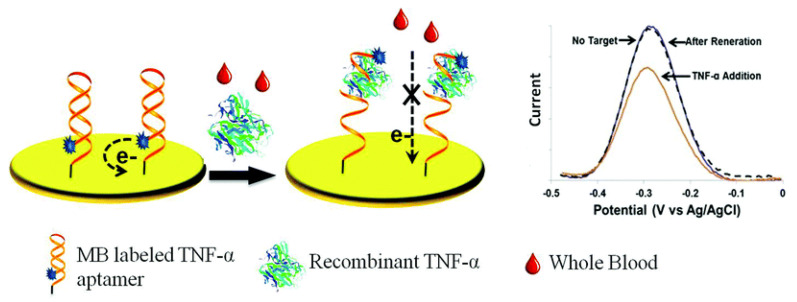
Schematic representation of an TNF-α aptasensor with methylene blue (MB) as a redox label. In the absence of the target, Apt hairpins remain folded with MB reporters in proximity to the electrode, ensuring efficient electron transfer and a measurable faradaic current. Upon target binding, the redox tag moves far away from the electrode and the redox current decreases. Reprinted from [106] with permission from The Royal Society of Chemistry.

Aptasensors employ aptamers (Apts), which are short, single-stranded oligonucleotides, as biorecognition elements. Compared with antibodies, Apts are an attractive candidate for biorecognition, owing to the ease of in vitro synthesis, modification and stability over a range of temperature and pH conditions [103]. Tertis et al. developed an impedimetric aptasensor for IL-6 detection consisting of anti-IL-6 Apts immobilized on polypyrrole and gold nanoparticle (AuNP)-coated screen printed carbon electrodes (SPCEs). Polypyrrole is an electroactive polymer with a low polymerization potential and, in combination with AuNPs, exhibits excellent biocompatibility. AuNPs provide a large surface area for Apt immobilization, thereby enhancing electron transfer [104]. This study showed how the immobilization of negatively charged oligonucleotides (i.e., Apt) on AuNPs can be accomplished much faster when coupled with a pulsed, externally applied oscillating potential compared with a constant applied potential [94]. Kumar et al. demonstrated an impedimetric aptasensor, which was used for measurements of IL-6 in sweat and serum. This sensor consisted of AuNPs and thiolated Apts immobilized on an Au electrode [105]. Liu et al. developed an electrochemical aptasensor using a methylene blue (MB)-conjugated Apt specific for TNFα onto an Au electrode. At room temperature, these Apts formed a hairpin structure, bringing the 5’ conjugated MB very close to the electrode, enabling passage of faradaic currents (Figure 3). Upon TNFα binding, the Apt straightens causing the MB to be situated far away from the electrode surface, reducing the passage of current in a proportionate manner [106].

**Figure 4:**
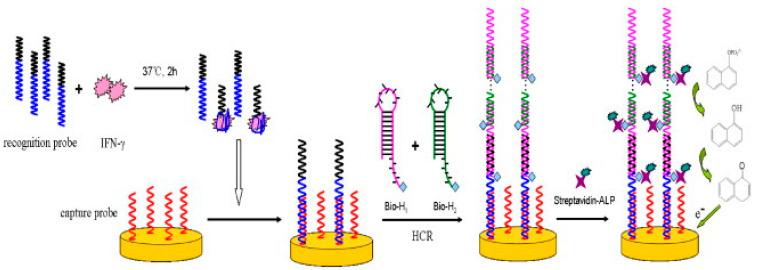
Schematic illustration of an electrochemical DNA biosensor for IFNγ based on hybridization chain reaction with enzyme-amplification. IFNγ partly quenches the recognition probes, while unbound recognition probes hybridize with the capture probes, followed by successive hybridization with biotinylated DNA hairpins. Tagged ALP dephosphorylates 1-NPP, which is further oxidized to 1-NP. Reprinted from [108] with permission from Elsevier.

Using a similar approach, Liu et al. developed an aptasensor comprised of 5’ MB-conjugated anti-IFNγ Apt immobilized on an Au electrode for IFNγ detection. The Apt-IFNγ bonds were disrupted using a buffer containing urea, and the sensor could be reused multiple times [107]. An aptasensor utilizing the unwinding mechanism of hairpins combined with four DNA sequences in successive partial hybridization was demonstrated by Zhao et al. DNA capture probes were immobilized on an Au electrode, and Apts specific to IFNγ were used as recognition probes. In the absence of IFNγ, Apts hybridized with the capture probes. However, IFNγ in the sample competitively bound and hindered this hybridization step. Subsequently, the surface immobilized, hybridized recognition probes induced the unwinding of the otherwise stable DNA hairpin H1 (biotinylated), followed by the hybridization of H1 to the sticky end of the recognition probes. This event further led to the unwinding and hybridization of DNA hairpin H2 (biotinylated) to the free end of H1. A cascade of successive hybridization events followed until H1 and H2 were quenched in the solution. Upon addition of streptavidin-conjugated alkaline phosphatase (ALP) and its substrate, 1-naphthyl phosphate (1-NPP), the latter was dephosphorylated to 1-naphthol (1-NP), which was irreversibly oxidized at the anode (Figure 4). The large assembly of ALP, generated by the induced cascade of hybridizations, resulted in high signal amplification [108]. Min et al. demonstrated an aptasensor for IFNγ detection by immobilizing DNA or RNA Apts on Au electrodes and compared their performance. Pentanethiol was used as a filler to ensure sufficient interspacing between Apts, so that the individual IFNγ proteins could bind to the Apt without hindrance. It was shown that RNA aptasensors could detect IFNγ at fM levels, while DNA aptasensors could only detect IFNγ in the pM range [109], presumably due to the different affinities of the Apts. 

An aptasensor consisting of MB-conjugated DNA hairpin Apt immobilized on Au electrodes for TGF-β1 detection was developed by Matharu et al. This biosensor was placed in a hepatic stellate cell culture, which was stimulated with platelet derived growth factor (PDGF) to produce TGF-β1 in the culture media. In order to prevent electrode fouling from the stellate cells, polydimethylsiloxane (PDMS) microcups were used as covering, which were only lifted during electrochemical measurements [110].

A summary of design and sensor preparation process as well as sensor performance parameters are presented on Table 1 and Table 2.

**Table 1:** Aptasensors: Design and sensor preparation.

Sl. No. Ref.	Transducer Components	Redox Species	Bio-Recognition Element	Blocking Agent	Preparation and Immobilization (Brief)
1. [94]	SPGE/PPyNPs/AuNPs/Apt	[Fe(CN)_6_]^4−/3−^	Anti-IL-6 Apt	6-mercaptohexanol	SPGE+ Py+ LiClO_4_ → polymerization: multipulse amperometry SPGE/PPyNPs+ HAuCl_4_+ H_2_SO_4_ → Au^3+^ reduction, NP formation: cyclic voltammetry SPGE/PPyNPs/AuNPs + Apt (thiolated): Au-S chemistry, multipulse amperometry SPGE/PPyNPs/AuNPs/Apt + MCH: Au-S chemistry, multipulse amperometry
2. [105]	Apt/AuNP/Au electrode	[Fe(CN)_6_]^4−/3−^	Anti-IL-6 Apt	HS-(CH_2_)_11_(OCH_2_CH_2_)_3_OH	Au electrode/AuNP+ Aptamer(thiolated): Au-S chemistry EG3+ Au electrode/AuNP/Aptamer: Au-S chemistry
3. [106]	Au/Apt-MB	MB (ox/red)	Anti-TNFα Apt	6-mercapto-1-hexanol	28-mer RNA Apt nucleotides are phosphorothiolated to protect from RNase 5’ NH_2_-Apt-(CH_2_)_6_SH 3’ + MB-NHS → 5’ MB-Apt-(CH_2_)_6_SH 3’ or Apt-MB Apt-(CH_2_)_6_S-S-(CH_2_)_6_-OH 3’ + TCEP+ H_2_O → Apt-(CH_2_)_6_SH+ HS-(CH_2_)6-OH + TCEP = O 5’ MB-Apt-(CH_2_)_6_SH 3’ + Au electrode: Au-S chemistry
4. [107]	Au/Apt-MB	MB (ox/red)	Anti-IFNγ Apt	6-mercapto-1-hexanol	3’ NH_2_-(CH_2_)_6_-Apt+ MB-NHS → 3’ MB-NH-(CH_2_)_6_-Apt or MB-Apt 5’ OH-(CH_2_)_6_-S-S-(CH_2_)_6_-Apt+ TCEP → 5’ HS-(CH_2_)_6_-Apt+ OH-(CH_2_)_6_-SH+ TCEP = O 5’ SH-Apt-MB 3’+ Au electrode: Au-S chemistry
5. [108]	Au/capture probe Recognition probe (Apt) H1-Bt H2-Bt SAv-ALP	1-naphthol (red → ox)	Anti-IFNγ Apt	6-mercapto-1-hexanol	Au+ 5’ SH-capture probe: immobilization with Au-S chemistry Recognition probe+ H1-Bt: hairpin opening, partial hybridization Partially hybridized Bt-H1-recognition probe+ H2-Bt: hairpin opening, partial hybridization; resulting in a cascade of successive hybridization events Bt-SAv: affinity bonding 1-naphthyl phosphate (1-NPP) converted to 1-naphthol (1-NP) by ALP
6. [109]	Au/Apt	[Fe(CN)_6_]^4−/3−^	Anti-IFNγ Apt	β-mercaptoethanol	Deactivation of RNase in solutions with diethyl pyrocarbonate (DEPC) Au electrode+ 5’ SH-Apt (DNA/RNA)+ dithiothreitol+ pentanethiol/MgCl_2_/PBS: Apt immobilization, interspaced with pentanethiol
7. [110]	Au/Apt-MB	MB(ox/red)	Anti-TGF-β1 Apt	6-mercapto-1-hexanol	MB-NHS+ 5’ NH_2_-Apt: covalent linkage; MB-Apt formation Au+ 3’ SH-Apt: Au-S chemistry 5’ NH_2_-Apt-SH 3’ (DNA) has a phosphorothioated backbone on 5’ adenine & cytosine nucleotides, to resist nuclease degradation

Common abbreviations: SPGE = Screen printed graphite electrode, PPyNP = Poly pyrrole nanoparticles, AuNP = Gold nanoparticles, H1, H2 = DNA hairpins, Bt= Biotin, SAv = Streptavidin, ALP = Alkaline Phosphatase, MB = methylene blue; Apt = Aptamer; MB-NHS = methylene blue, carboxylic acid, succinimidylester, EG3 = HS-(CH_2_)_11_(OCH_2_CH_2_)_3_OH, an oligo(ethylene oxide), TCEP = tris-(2-carboxyethyl)phosphine hydrochloride.

**Table 2:** Aptasensors: Specifications and performance characteristics.

Sl. no. Ref.	Detection Technique	Limit of Detection	Range of Detection	Interfering Species Tested	Incubation Time	Sample Type	Reproducibility	Stability	Repeatability
1. [94]	EIS	0.33 pg/mL	1 pg/mL–15 μg/mL	BSA, CEA, MUC1, MUC4, MUC16	+IL-6/30 min	IL-6 in PBS Spiked serum	(6 assays) RSD = 3.42%	-	-
2. [105]	EIS	0.02 pg/mL	0.02–20 pg/mL	BSA	-	IL-6 in buffer Spiked artificial sweat	(5 assays, at [IL-6] = 0.02 pg/mL) RSD = 14.1%	After 2 weeks- 90% of initial signal retained for [IL-6] = 0.2 pg/mL	90% of initial signal retained for [IL-6] = 0.02 pg/mL
3. [106]	SWV	58 pM	58 pM–6 nM	-	Sensor equilibration/30 min +TNFα/15 min	rTNFα in spiked whole blood	-	Stable over 10 h	After 6 cycles & regeneration/urea, 90% of original signal retained
4. [107]	SWV	0.06 nM	0.06–10 nM	IgG, anti-IgG, BSA	+IFNγ/15 min	rIFNγ in HEPES buffer IFNγ in RPM1 culture media IFNγ in RPM1/serum	-	-	Regeneration/urea/1min + rinsing/diH2O; sensor can be reused more than 10 or more times
5. [108]	DPV	0.3 nM	0.5–300 nM	PDGF-BB, BSA, IgG, CEA, IL-6	IFNγ+ recognition probe/2 h +Au/capture probe/1 h +H1-Bt, H2-Bt/90 min, rinsing/10 min +SAv-ALP/30 min, rinsing/20 min +1NPP/3 min	IFNγ in HEPES buffer IFNγ in RPM1 culture media IFNγ in RPM1/serum	-	After 2 weeks, no significant change in current response	Regeneration/NaOH/20 min + washing/diH_2_O/10 min; sensor can be reused more than 3 times
6. [109]	EIS	500 fM (RNA Apt) 1 pM (DNA Apt), 1.21 pM (DNA in FBS)	-	BSA, FBS	+IFNγ/30 min	IFNγ in PBS Spiked FBS	-	-	-
7. [110]	SWV	1 ng/mL	1–200 ng/mL	IL-2, IFNγ, BSA, IgG, TGF-β2, TGF-β3	(real-time detection at constant flow)	rTGF-β1 in cell culture media DMEM	-	-	-

Common abbreviations: PBS = Phosphate-buffered saline, BSA = Bovine Serum Albumin, CEA = Carcinoembryonic antigen, MUC = Mucin protein, Ig = Immunoglobulin protein, PDGF = Platelet-derived growth factor, FBS = Fetal bovine serum, HEPES = (4-(2-hydroxyethyl)-1-piperazineethanesulfonic acid), DMEM = Dulbecco’s Modified Eagle Medium.

#### 2.1.2. Enzyme-Labeled Immunosensors

**Figure 5:**
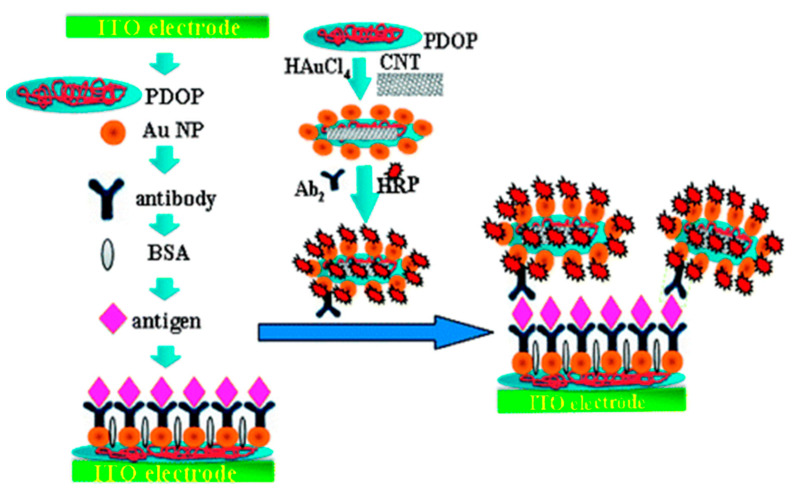
Preparation of ITO/PDOP-AuNP/Ab_1_ platform and HRP-Ab_2_-AuNP-PDOP@CNT Probe and sandwich type immunoassay. IL-6 binds to Ab_1_ and Ab_2_ and immobilizes HRP, which oxidizes 1,2-phenylenediamine in the presence of H_2_O_2_. Reprinted from [111] with permission from American Chemical Society.

Enzymatic immunosensors are one of the most widely explored types of electrochemical biosensors for cytokine detection, where an enzyme is used as a reporter molecule, which catalyzes the conversion of a substrate to product by reducing its activation energy. Horseradish peroxidase (HRP) is one of the most commonly used enzyme reporters due to its stability, small size and high turnover rate. In a study published by Wang et al., an amperometric sandwich-type immunosensor was developed for the detection of IL-6 using an anti-IL-6 capture antibody (Ab_1_) and anti-IL-6 signal antibody (Ab_2_). Ab_1_ was immobilized on a surface modified indium tin oxide (ITO) electrode, decorated with polydopamine (PDOP) and AuNPs. Ab_2_ was attached to carbon nanotubes (CNTs) functionalized with PDOP and AuNPs. HRP adsorbed on the CNTs catalyzed the oxidation of 1,2-phenylenediamine in the presence of H_2_O_2_, followed by cathodic reduction which generated the detection signal (Figure 5) [111]. CNTs exhibit favorable properties for electrochemical detection such as enhanced electron transfer kinetics, excellent conductivity and a large surface area to volume ratio, available for functionalization [112]. Munge et al. prepared and compared two biosensor platforms where CNTs and AuNPs were posed against each other. One sensor was functionalized with glutathione capped AuNPs, and the other sensor was coated with single-walled carbon nanotubes (SWCNTs) forests. The LOD of the former (10 pg/mL) was 3 times lower than the latter (30 pg/mL). For the AuNP sensor, AuNPs were immobilized on a poly(diallyldimethylammonium chloride) (PDDA) functionalized pyrolytic graphite electrode, with covalently attached anti-IL-6 Ab_1_. The antigen binding specificity was enhanced using biotinylated Ab_2_ (Bt-Ab_2_). In the presence of IL-6, streptavidin conjugated HRP (SAv-HRP) was attached to the sensor surface, generating an amperometric current with the redox cycling of hydroquinone in the presence of H_2_O_2_ [95]. 

Zhang et al. developed an immunosensor for IFNγ detection by utilizing AuNPs as transducers, coated with PDDA and Ab_1_, attached to ITO electrodes. AuNPs also formed the basis of bionanolabels, coated with Bt-Ab_2_ and SAv-HRP enzyme label, and hydroquinone and H_2_O_2_ were used as substrates [113]. Bt-Ab_2_ and SAv-HRP labels were also used for the construction of a sandwich type immunosensor by Sanchez-Tirado et al., wherein SPCE grafted with electrochemically reduced p-aminobenzoic acid (p-ABA) diazonium salt served as a transducer, to which anti-IFNγ Ab_1_ was attached [114].

**Figure 6:**
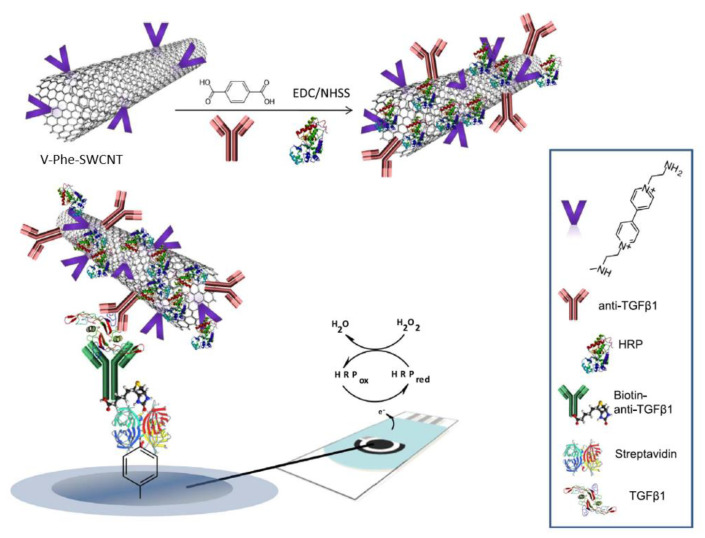
Schematic illustration of the different steps involved in the construction of an amperometric immunosensor for TGF-β1 using V-Phe-SWCNT hybrids. Ab_1_, HRP and a viologen were immobilized on SWCNT. Ab_2_ was immobilized via Bt-SAv on a screen printed carbon electrode (SPCE) grafted with carboxyl aryl diazonium. The signal generated by HRP catalyzed H_2_O_2_ reduction was amplified by the redox mediator viologen. Reprinted from [116] with permission from Elsevier.

The first amperometric biosensor for measuring TGF-β1 was developed by Sanchez-Tirado et al., which employed molecular beads functionalized with Ab_1_ using a Mix & Go polymer, Bt-Ab_2_ and a SAv-HRP (catalyst)-hydroquinone, H_2_O_2_ (substrates) system. This sensing platform could detect TGF-β1 in plasma and urine samples at concentrations of 0.1–25 ng/mL and 10–50 pg/mL, respectively [115]. Two other amperometric immunosensors for TGF-β1 detection were reported using alternative approaches for antibody immobilization. The first approach, demonstrated by Sanchez-Tirado et al., employed a free radical grafting method with carboxyl aryl diazonium on screen printed carbon electrodes (SPCEs) containing SWCNTs, similar to the work described in [114]. Ab_2_ was immobilized onto the SPCE via Bt-SAv, and Ab_1_ and HRP were immobilized onto SWCNTs, which also contained 1-(3-aminoethyl)-4,4’-bipyridinium bromide, a viologen, as a redox mediator to amplify the redox signal generated by hydroquinone and H_2_O_2_ (Figure 6). Viologens are known to have three oxidation states: +2, +1, 0 at negative potentials; the first two oxidation states are utilized here to shuttle electrons between the electrode and proteins [116]. The second approach for antibody immobilization involved azide-alkyne cycloaddition between azide-functionalized multi-walled carbon nanotubes (MWCNTs) and ethynylated fragment crystallizable (F_c_) region of anti-Ab_1_ IgG, in the presence of Cu(I) catalyst. Developed by Sanchez-Tirado et al., this approach also employed Bt-Ab_2_ and a SAv-HRP (catalyst)-hydroquinone, H_2_O_2_ (substrate) system. 11-Azide-3,6,9-trixaundecan-1-amine was used for azide (-N_3_ gr.) functionalization, and sodium periodate (NaIO_4_) was used to oxidize 1,2-diols of F_c_ region of carbohydrates into alkyne groups [117].

**Figure 7:**
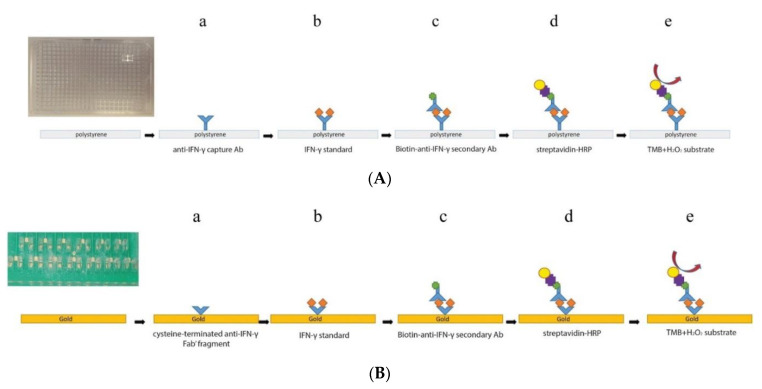
Schematic of (**A**) IFNγ ELISA assay followed in 96- or 384-well plates and (**B**) IFNγ amperometric assay followed in gold Au/PMMA/PCB plates. Oxidization of TMB_red_ by HRP induces photometric change measured in ELISA, and a reduction current of TMB_ox_ is measured in the amperometric biosensor. Reprinted from [118] with permission from Elsevier.

A relatively unique attempt was made by Moschou et al. to adapt an ELISA platform onto a printed circuit board (PCB) platform for amperometric measurements of IFNγ. Except for the replacement of the capture antibody (Ab_1_) by an antigen binding fragment (F_ab1_) of the capture antibody, the rest of the setup was identical, mounted onto an Au electrode for electrochemical transduction (Figure 7). In ELISA, a chromogenic 3,3’,5,5’-tetramethylbenzidine (TMB_red_) solution (bluish-green hue) is oxidized by HRP in the presence of H_2_O_2_, resulting in a 3,3’,5,5’-tetramethylbenzidine diimine (TMB_ox_) solution (blue hue). Adding an acid/stop reagent turns the solution yellow. These color changes are read by a spectrophotometer and correlated with the IFNγ concentration. In the amperometric biosensor, the reduction current of TMB_ox_ was used for IFNγ quantification. The calibration plot obtained was comparable to that of ELISA [118].

Alkaline phosphatase (ALP) is another enzyme commonly used as a reporter for electrochemical biosensors. Yin et al. proposed an ALP-containing bioconjugate label whereby poly(styrene-acrylic acid) (PSA) spheres were functionalized with polyallylamine hydrochloride (PAH). These spheres were also coated with AuNPs and anti-TNFα Ab_2_. Anti-TNFα Ab_1_ was covalently immobilized on a glassy carbon electrode (GCE) modified with poly(acrylic acid)-doped polyaniline, and 1-naphthyl phosphate was used as a substrate [119]. In addition to the use of an enzymatic label, polymers have been generously used in sensor fabrication, which furnish with an ample amount of a variety of functional groups, as well as high loading capacity. Arya et al. proposed a unique approach to biosensor fabrication by immobilizing anti-TNFα Ab_1_ away from the electrode surface to avoid degradation or alteration of antibodies upon the application of an external bias potential, referred to as an off-matrix modified sensor. This matrix was prepared by punching laser-engraved wells on polymethyl methacrylate (PMMA) sheets, which were then coated with Ab_1_ via a 4-fluoro-3-nitroazidobenzene linker and attached to Au comb electrodes. TNFα samples, Bt-Ab_2_-AuNP and SAv-ALP were added to the sensor through two fluidic channels, and 4-aminophenylphosphate was used as a substrate [120]. Arya and Estrela further optimized this approach by using carboxyl polypyrrole films on a comb-shaped Au electrode microarray to detect TNFα, which could be measured at concentrations as low as 78 pg/mL in undiluted serum samples [121].

**Figure 8:**
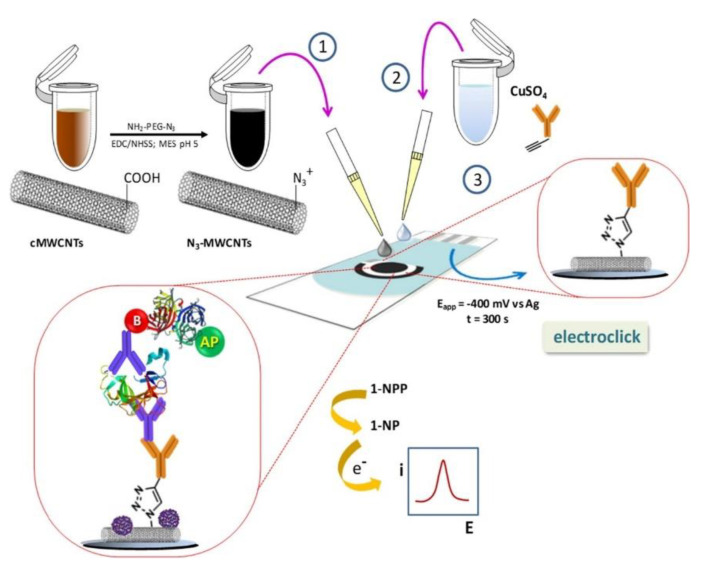
Schematic illustration of the steps involved in the preparation of the SPCE/MWCNT-IgG-Ab_1_-IL-1β-Ab_2_-Bt-SAv-ALP immunosensor by electro-click chemistry, where MWCNT is multi-walled carbon nanotubes. Ethynylated anti-Ab_1_ IgG is attached to azide-functionalized MWCNTs in the presence of Cu(I). Upon sandwich immunoreaction, ALP dephosphorylates 1-naphthyl phosphate to 1-naphthol, which is oxidized at the electrode. Reprinted from [123] with permission from Elsevier.

Guerrero et al. developed an immunosensor for the detection of IL-1β, a 17.5 kDa protein, in saliva samples [122]. They used azide-alkyne cycloaddition (electro-click chemistry) in the presence of a Cu(I) catalyst for the immobilization of ethynylated anti-Ab_1_ IgG on azide-functionalized MWCNTs, similar in principle to the TGF-β1 sensor described in [117]. Bt-Ab_1_ and a SAv-ALP (catalyst)-1-naphthyl phosphate (substrate) system were utilized for voltammetric detection on an SPCE (Figure 8). This sensor claimed to offer an improved detection range (10–200 pg/mL) compared to ELISA (15.6–500 pg/mL), as well as shorter assay duration (2 h 30 min) compared with ELISA (3 h 45 min) [123].

**Figure 9:**
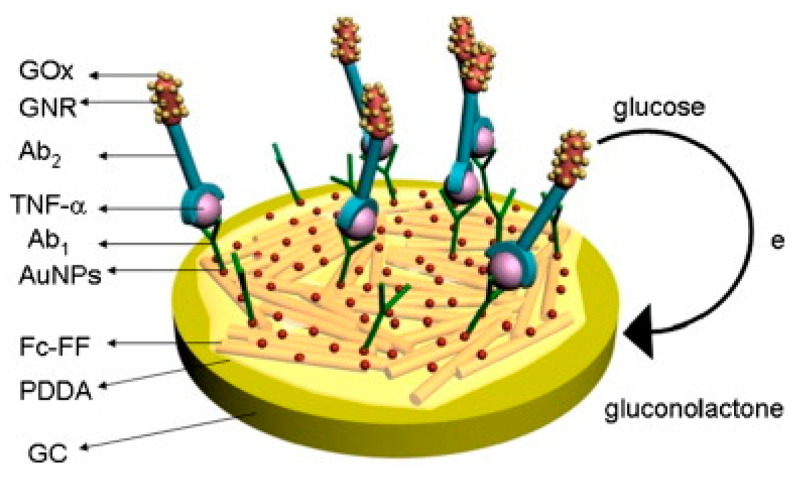
Schematic illustration of the GCE/Fc-PNW/PDDA/AuNP/Ab_1_, GNR-GOx-Ab_2_ immunosensor for TNFα detec Table 1. Ab_2_ functionalized with gold nanorods (GNRs) and glucose oxidase (GOx) are brought close to the electrode upon sandwich immunoreaction. GOx converts glucose to gluconolactone. Reprinted from [124] with permission from Elsevier.

Sun et al. developed a sandwich immunosensor for TNFα detection using glucose oxidase (GOx) as an enzyme label, which converts glucose to gluconolactone. This biosensor utilized a PDDA-coated Au electrode containing ferrocene (Fc) carboxylic acid-conjugated peptide nanowires (diphenylalanine), AuNPs and Ab_1_. Ab_2_ was functionalized with gold nanorods (GNRs) and glucose oxidase (GOx) via a cysteine–glutaraldehyde bridge (Figure 9). Peptide nanowires (PNWs) exhibit the properties of self-assembly, hydrophobicity and can load Abs in high proportions. The signal generated by glucose oxidation was greatly amplified by the immobilized ferrocene mediators [124].

A summary of design and sensor preparation process as well as sensor performance parameters are presented on Table 3 and Table 4.

**Table 3:** Enzyme-labeled immunosensors: Design and sensor preparation.

Sl. no. Ref.	Transducer Components	Redox Species	Bio-Recognition Element	Blocking Agent	Preparation and Immobilization (Brief)
1. [111]	ITO/PDOP-AuNP/Ab_1_ HRP-Ab_2_-AuNP-PDOP@CNT	1,2-phenylenediamine/2,2’-diaminoazobenzene H_2_O_2_/H_2_O	Anti-IL-6 Ab_1_, Ab_2_	BSA	ITO + Dopamine + AuNP: polymerization of dopamine, AuNP absorption ITO/PDOP-AuNP + Ab_1_: physical absorption CNT + dopamine + HAuCl_4_: Polymerization of dopamine, formation & absorption of AuNP HRP+ Ab_2_-AuNP-PDOP@CN: Physical absorption
2. [95]	PG/PDDA/GSH-AuNP/Ab_1_/BSA Bt-Ab_2_ SAv-HRP	Hydroquinone/benzoquinone H_2_O_2_/H_2_O	Anti-IL-6 Ab_1_, Ab_2_		GSH + AuNP: Au-S chemistry GSH-AuNP + Ab_1_: amide bond formation with EDC/NHS catalyst Bt-Ab_2_+ SAv-HRP: affinity bonding
3. [113]	ITO/PDDA/AuNP/Ab_1_ AuNP-Ab_2_-Bt-SAv-HRP	Hydroquinone/benzoquinone H_2_O_2_/H_2_O	Anti-IFNγ Ab_1_, Ab_2_	BSA	ITO electrode + PDDA: drop-casting ITO/PDDA(+ve) + AuNP(-ve): electrostatic adsorption ITO/PDDA/AuNP + Ab_1_: adsorption Citrate reduction of HAuCl_4_: AuNP formation HRP-Ab_2_+ AuNP: adsorption Bt+ SAv: affinity bonding
4. [114]	SPCE-Phen-Ab_1_ Bt-Ab_2_ SAv-HRP	Hydroquinone/benzoquinone H_2_O_2_/H_2_O	Anti-IFNγ Ab_1_, Ab_2_	BSA	p-aminobenzoic acid (p-ABA) + NaNO_2_/HCl → p-ABA diazonium salt SPCE + p-ABA diazonium salt: electrochemical reduction; grafting with CV SPCE-Phe-COOH + Ab_1_: amide bond formation with EDC/NHS catalyst
5. [115]	Mb-Ab_1_ Bt-Ab_2_ SAv-HRP- SPCE	Hydroquinone/benzoquinone H_2_O_2_/H_2_O	Anti-TGF-β1 Ab_1_, Ab_2_	Ethanolamine	MB-COOH+ Mix&Go polymer + Ab_1_: covalent immobilization SAv+ Bt: affinity bonding MB-Ab_1_-TGFβ-1-Ab_2_-HRP separated with magnet at the base of SPCE
6. [116]	SPCE/SAv-Bt-Ab_2_ V-Phen-SWCNT(-HRP)-Ab_1_	(C_5_H_4_N(CH_2_)_2_NH_2_)^1+/2+^ or V^1+/2+^ H_2_O_2_/H_2_O Hydroquinone/benzoquinone	Anti-TGF-β1 Ab_1_, Ab_2_	Biotin	SWCNT + p-aminobenzoic acid (p-ABA) + isoamylnitrile/NMP → SWCNT-Phe-COOH; grafting 4,4’-bipyridine+ 2-bromoethylamine/CH_3_CN → 1-(3-aminoethyl)-4,4’-bipyridinium bromide ((C_5_H_4_N(CH_2_)_2_NH_2_)_2_^2+^), a viologen V (C_5_H_4_N(CH_2_)_2_NH_2_)_2_^2+^ + SWCNT-Phe-COOH → SWCNT-Phe-V; amide bond formation with DCC/HOBt catalyst SWCNT-Phe-V + Ab_1_, HRP: amide bond formation with EDC/NHSS catalyst SPCE+ p-ABA+ NaNO_2_/HCl → SPCE-Phe-COOH, diazotization followed by reduction, grafting with CV SPCE-Phe-COOH + SAv: amide bond formation with EDC/NHSS catalyst SAv + Bt: affinity bonding
7. [117]	SPCE/MWCNT-alkyne-azide-IgG Ab_1_ Bt-Ab_2_ SAv-HRP	Hydroquinone/benzoquinone H_2_O_2_/H_2_O	Anti-TGF-β1 Ab_1_, Ab_2_	Casein	MWCNT(-COOH gr.) + 11-azide-3,6,9-trixaundecan-1-amine: amide bond formation with EDC/NHS catalyst IgG+ NaIO_4_: oxidation of 1,2-diols of glycosylated Fc region of Ab, leading to alkyne formation MWCNT(-N_3_ gr.) + IgG(-C≡CH gr.): azide-alkyl cycloaddition into triazole, with ascorbic acid+ Cu(I) catalyst; click chemistry MWCNT-alkyne-azide-IgG drop-casted on SPCE
8. [118]	PMMA/PCB/Au/F_ab1_-cys Bt-Ab_2_ SAv-HRP	3,3’,5,5’-tetramethylbenzidine (TMB_ox_/TMB_red_) H_2_O_2_/H_2_O	Anti-IFNγ Fab_1_, Ab_2_	BSA	PMMA/PCB containing Au plated microchannels+ F_ab_-cys: Au-S chemistry Bt+ SAv: affinity bonding TMB oxidation by HRP, coupled to H_2_O_2_ reduction
9. [119]	GCE/PANA/Ab_1_ PSA/PAH/AuNP-Ab_2_-ALP	α-naphthol (1-NP) (red→ox)	Anti-TNFα Ab_1_, Ab_2_	BSA	Styrene + acrylic acid + K_2_S_2_O_8_: polymerization into PSA spheres PSA+ PAH: functionalization PSA/PAH(+ve) + AuNP(-ve) colloid: electrostatic attraction Aniline + PAA: electropolymerization into PANA on GCE GCE/PANA + Ab_1_; amide bond formation with EDC/NHS catalyst α-naphthyl phosphate (1-NPP) converted to α-naphthol by ALP
10. [120]	Si/SiO_2_/Au/PMMA/FNAB/Ab_1_ Bt-Ab_2_-AuN SAv-ALP-AuNP	4-aminophenol (4AP)/quinoneimine(QI)	Anti-TNFα Ab_1_, Ab_2_	StartingBlock T20 (PBS) blocking buffer	PMMA sheet: laser-engraved, with fluidic channels punched PMMA+ FNAB: attachment with amine bond formation PMMA/FNAB+ Ab_1_: attachment with amine bond formation 4-aminophenyl phosphate (4APP) converted to 4AP by ALP Comb-shaped Au electrodes fabricated on Si/SiO_2_ wafers
11. [123]	SPCE/MWCNT-IgG Ab_1_ Bt-Ab_2_ SAv-ALP	1-naphthol (red→ox)	Anti-IL-1β Ab_1_, Ab_2_	Caesin	CuSO_4_: Cu(II)→Cu(I); electrochemical reduction MWCNT(-COOH gr.) + 11-azide-3,6,9-trixaun-decan-1-amine: amide bond formation IgG + NaIO_4_: oxidation of 1,2-diols of glycosylated Fc region of Ab, leading to alkyne formation MWCNT drop-casted on SPCE SPCE/MWCNT(-N_3_ gr.) + IgG(-C≡CH gr.): azide-alkyl cycloaddition into triazole, with Cu(I) catalyst; electro-click chemistry 1-naphthyl phosphate (pNPP) converted to 1-naphthol (pNP) by ALP
12. [124]	GCE/Fc-PNW/PDDA/AuNP/Ab_1_ GNR-GOx-Ab_2_	Fe^2+/3+^ of Ferrocene Glucose/gluconolactone	Anti-TNFα Ab_1_, Ab_2_	BSA	Boc-Phe-Phe-OH + H-Phe-OMe + HBTU/HOBT/CH_2_Cl_2_, Et_3_N → Boc-Phe-Phe-OMe; separated by column chromatography Boc-Phe-Phe-OMe + CF_3_COOH/CH_2_Cl_2_ → H-Phe-Phe-OMe H-Phe-Phe-OMe+ Et_3_N/CH_2_Cl_2_ + Fc-OBt → Fc- Phe-Phe-OMe Fc-Phe-Phe-OMe+ CF_3_CHOH/CH_3_OH → Fc-Phe-Phe-OH; self-assembly to Fc-PNW CTAB + HAuCl_4_+ NaBH_4_: Au reduction; + CTAB+ HAuCl_4_ + AgNO_3_+ C_6_H_8_O_6_: GNR formation GNR+ cysteine(SAM)+ glutaraldehyde + GOx, Ab_2_: covalent immobilization Fc-PNW coated with PDDA, AuNP & Ab_1_ adsorbed

Common abbreviations: PDOP = polydopamine, PG = Pyrolytic graphite, GSH = Glutathione, PDDA = poly(diallyldimethylammonium chloride), Phen = Phenyl gr., Mb = Magnetic bead, V = Viologen (C_5_H_4_NR)_2_^2+^ where R = modified alkyl group, CNT = Carbon nanotube, SWCNT = Single-walled CNT, MWCNT = Multi-wall CNT, PMMA = Polymethyl methacrylate, PCB = Printed circuit board, F_ab1_ = antigen binding fragment, cysteine terminated, PANA = polyaniline doped with poly(acrylic acid), PSA = poly(styrene-acrylic acid), PAH = polyallylamine hydrochloride, PMMA = Polymethyl methacrylate, FNAB = 4-fluoro-3-nitroazidobenzene, Fc = ferrocene, PNW = peptide nanowires/diphenylalanine, GNR = gold nanorods, AuNP = Gold nanoparticles, ITO = Indium tin oxide, GCE = Glassy carbon electrode, SPCE = Screen printed carbon electrode, ALP = alkaline phosphatase, HRP = horseradish peroxidase, GOx = Glucose oxidase, Bt = Biotin, Av = Avidin, SAv = Streptavidin; Ab_1_ = capture antibody, Ab_2_ = signal antibody; EDC = 1-ethyl-3-(3-dimethylaminopropyl)carbodiimide, DCC = N,N’-dicyclohexylcarbodiimide, NHS = N-hydroxysuccinimide, NHSS = N-hydroxysulfo-succinimide, HOBt = Hydroxybenzotriazole, PAA = poly(acylic acid), Boc = Butoxycarbonyl gr., Phe = Phenylalanine, Me = Methyl gr., HBTU = Hydroxybenzotriazole, HOBT = 2-(1H-bemzotriazole-1yl)-1,1,3,3-tetramethyluronium hexafluorophosphate, Et_3_N = Trimethylamine, CTAB = Cetyltriethylammonium bromide.

**Table 4:** Enzyme-labeled immunosensors: Specifications and performance characteristics.

Sl. no. Ref.	Detection Technique	Limit of Detection	Range of Detection	Interfering Species Tested	Incubation Time	Sample Type	Reproducibility	Stability	Repeatability
1. [111]	CA	1.0 pg/mL	4–800 pg/mL	AFP, CEA, hIgG, L-cys, L-lys, Glucose	+IL-6/1h +HRP-Ab_2_-AuNP-PDOP@CNT/50 min	IL-6 in buffer Serum sample	Intra-assay precision (5 readings per run, at [IL-6] = 40 pg/mL) SD = 5.5% Inter-assay precision (5 sensor samples, at [IL-6] = 40 pg/mL) SD = 6.8%	After 30 days- 85.5% of initial signal retained	-
2. [95]	Rotating disc amperometry	10 pg/mL	10–4000 pg/mL	-	+IL-6/1 h +Bt-Ab_2_/1 h +SAv-HRP/30 min	Recombinant human IL-6 (rhIL-6) in calf serum	Sensitivity: 1.6 nA/cm^2^ (pg/mL IL-6)		
3. [113]	DPV	0.048 pg/mL	0.1–10,000 pg/mL	BSA, AA, glucose, UA, IL-22	+IFNγ/2 h +AuNP-Ab_2_-HRP/1 h	IFNγ in PBS Serum sample	(5 assays, at [IFNγ] = 0.1 ng/mL) RSD = 2.7%	After 2 weeks, 92% of initial signal retained	(disposable)
4. [114]	CA	1.6 pg/mL	2.5–2000 pg/mL	AA, BSA, Glucose, Hb, hIgG, IL-1β, IL-6, IL-8, RANKL, TGF-β1, TNFα, UA	+IFNγ/60 min +Bt-Ab_2_/45 min +SAv-HRP/20 min	IFNγ in PBS Biological international standard (BIS) for IFNγ Saliva sample	(5 assays, same day, at [IFNγ] = 1 ng/mL) RSD = 1.8% (5 assays, different days, at [IFNγ] = 1 ng/mL) RSD = 2.6%	Within 40 days, retained signal acceptable	-
5. [115]	CA	10 pg/mL	15–3000 pg/mL	AA’, UA, Creatinine, APN, IL-6, IL-8, TNFα	+TGF-β1/60 min +Bt-Ab_2_, BSA/60 min +SAv-HRP/20 min	TGF-β1 in ELISA standard solution Spiked urine	(5 assays, same day, at [TGF-β1] = 250 pg/mL) RSD = 3.9% (5 assays, different days, at [TGF-β1] = 250 pg/mL) RSD = 4.2%	Signal within control limits within 30 days	-
6. [116]	CA	0.95 pg/mL	2.5–1000 pg/mL	AA’, APN, BSA, Cortisol, IgG, IL-1β, IL-6, IL-8, TNFα, UA	+TGF-β1/60 min +V-Phe-SWCNT(-HRP)-Ab_1_/60 min	TGF-β1 in ELISA standard solution Saliva sample	(5 assays, same day, at [TGF-β1] = 125 pg/mL) RSD = 3.1% (5 assays, different days, at [ TGF-β1] = 125 pg/mL) RSD = 7.2%	Signal within control limits for: (i) SPCE/SAv-Bt-Ab_2_, within 30 days; (ii) V-Phe-SWCNT(-HRP)-Ab_1_, within 14 days	-
7. [117]	CA	1.3 pg/mL	5–200 pg/mL	APN, BSA, BR, CP, TNFα, GHRL, Hb, IL-6, IL-8, CRP, Chl, TGF-β2, TGF-β3, TGF-β5	+TGF-β1, Bt-Ab_2_/60 min +SAv-HRP/20 min	TGF-β1 in ELISA standard solution Spiked human serum	(5 assays, same day, at [TGF-β1] = 125 pg/mL) RSD = 2.7% (5 assays, different days, at [TGF-β1] = 125 pg/mL) RSD = 2.5%	Signal within control limits within 40 days	-
8. [118]	CA	126.75 pg/mL	15–1000 pg/mL	TREM-1	+F_ab1_-cys/60 min + IFNγ standard+ Bt-Ab_2_/1 h +SAv-HRP/20 min +TMB, H_2_O_2_/20 min	IFNγ in ELISA standard solution Spiked human serum	-	-	-
9. [119]	DPV	0.01 ng/mL	0.02–200 ng/mL	CEA, BSA, hIgG	+TNFα/1 h +PSA/PAH/AuNP-Ab_2_-ALP/1 h +pNPP/10 min	TNFα in buffer Human serum sample	Intra-assay precision (7 runs, at [TNFα] = 5 ng/mL) RSD=5.7% Inter-assay precision (7 assays, at [TNFα] = 5 ng/mL) RSD = 8.1%	After 1 month, 95% of initial response retained	-
10. [120]	DPV	66.8 pg/mL	0.1–100 ng/mL	IL-2, IFNγ	+TNFα/20 min +Bt-Ab_2_/20 min +SAv-ALP/20 min +4APP/20 min	TNFα in spiked undiluted serum	-	Until 6 weeks, 96% of initial signal was retained, after which it dropped to 90% by 9th week	-
11. [123]	DPV	5.2 pg/mL	1st slope: 10–200 pg/mL; 2nd slope: 200–1200 pg/mL	BR, TGF-β1, LEP, IL-8, IL-6, Hb, Chl, BSA, GHRL, TNFα	+IL-1β/1 h +Bt-Ab_2_/1 h +SAv-ALP/20 min +1-NPP/5 min	IL-1β standard solution Spiked saliva	(10 assays, same day, at [IL-1β] = 1 ng/mL) RSD = 5.2% (10 assays, different days, at [IL-1β] = 1 ng/mL) RSD = 6.2%	Signal within control limits within 10 days	(disposable)
12. [124]	SWV	2 pg/mL	0.005–10 ng/mL	-	+TNFα/1 h +GNR-GOx-Ab_2_/1 h	TNFα in buffer Spiked serum	(6 assays, at [TNFα] = 0.1 ng/mL) RSD = 5.4%	-	-

Common abbreviations: PBS = Phosphate-buffered saline, BSA = Bovine Serum Albumin, CEA = Carcinoembryonic antigen, hIg = Human immunoglobulin protein, AFP = α-1-fetoprotein, CRP = C-reactive protein, L-cys = L-cysteine, L-lys = L-lysine, AA = Abscisic acid, UA = Uric acid, RANKL = Receptor activator of nuclear factor kappa-B ligand, AA’ = Ascorbic acid, APN = Adiponectin, BR = Bilirubin, CP = Cerculoplasmia, GHRL = Ghrelin, Hb = Hemoglobin, Chl = Cholesterol, LEP = Leptin, TREM1 = Triggering receptor expressed on myeloid cells 1.

#### 2.1.3. SAM-Enabled Impedimetric Immunosensors

Impedimetric immunosensors consist of a transducing electrode coated with a biorecognition element (Ab or Apt) where binding of the target protein results in a change in the electrical impedance. One of the main advantages of impedimetric immunosensors is that they are label free and do not require an enzyme label or reporter molecule, thereby simplifying the detection process. Yang et al. developed an impedimetric immunosensor for IL-6 detection consisting of a Si/SiO_2_ electrode modified with SWCNTs and AuNPs immobilized with anti-IL-6 Ab. A combination of SWCNTs and AuNPs exhibited efficient and enhanced electron transfer rate. Using this immunosensor, IL-6 could be detected at concentrations as low as 0.01 fg/mL [125]. 

**Figure 10:**
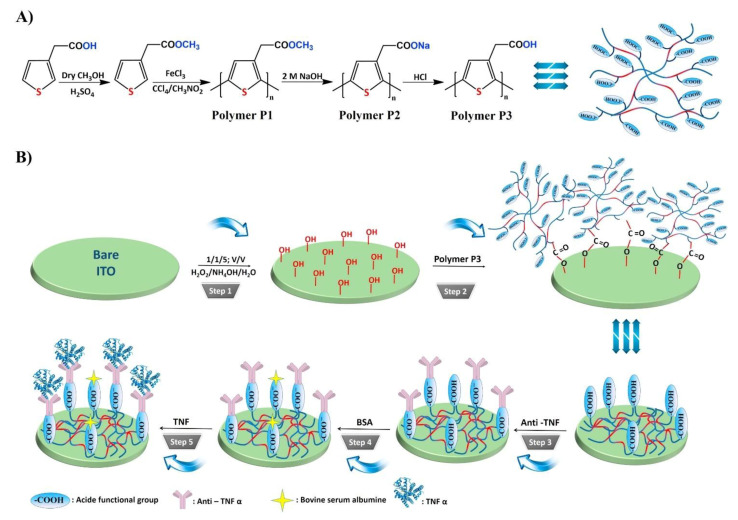
Schematic illustration depicting the bio-functionalization steps of a ITO/P3/TNFα biosensor. (**A**) Process for generating P3 polymer. (**B**) Ab is immobilized on the ITO electrode via P3 linker, which captures TNFα. Reprinted from [96] with permission from Elsevier.

Bellagambi et al. developed an impedimetric immunosensor for the detection of TNFα in human saliva. Anti-TNFα Ab was immobilized on an Au electrode using a 4-carboxymethyl aniline linker. Measurements were performed using spiked samples with known TNFα concentrations to generate a calibration curve, which was used for measurements of human saliva samples. Using this sensor, TNFα could be detected in human saliva within the range of 1–15 pg/mL [126]. Aydin et al. constructed an impedimetric immunosensor for TNFα detection consisting of a hydroxylated ITO electrode functionalized with a semiconducting poly(3-thiophene acetic acid) linker. The poly(3-thiophene acetic acid) modification process resulted in carboxylic acid groups on the surface, facilitating the immobilization of anti-TNFα Ab (Figure 10). Measurements in clinical saliva samples revealed that this sensor could detect TNFα at concentrations as low as 3.7 fg/mL [96].

Dijksma et al. demonstrated an impedimetric immunosensor for IFNγ detection, where anti-IFNγ Ab was immobilized on an Au electrode using a SAM of N-acetylcysteine (NAC). Both impedimetric and amperometric measurements were performed, and impedimetric quantification was found to yield a lower LOD. The authors utilized cumulative out-of-phase impedance (Σ∆Z”) shift as the calibration parameter, instead of charge transfer resistance (R_ct_) (utilized more frequently), and the measurements were taken with multiple successive flow-injections at a constant flow-rate [127]. Yao et al. developed an immunosensor using polyethylene glycol (PEG)-linked Ab onto interdigitated Au electrodes for the impedimetric detection of TGF-β1 [128]. Baraket et al. developed an impedimetric immunosensor for IL-10 detection, which is a potential biomarker for estimating the extent of inflammation post left ventricular assisted device (LVAD) implantation in end-stage heart failure patients [129]. This immunosensor utilizes carboxyl diazonium-free radical grafting on an Au-polyimide electrode for anti-IL-10 Ab immobilization [130]. Another IL-10 biosensor developed by Lee et al. employed a hafnium oxide (HfO_2_) functionalized Si substrate, where Ab immobilization was facilitated using a 11-(triethoxysilyl)undecanal (TESUD) SAM. Using this immunosensor, IL-10 could be detected at concentrations as low as 0.1 pg/mL [131].

Pui et al. devised an integrated system combining an impedimetric sensor with cytokines-producing cell cultures for TNFα quantification via EIS. Anti-TNFα Ab was linked to Au electrodes (in arrays) using a reduced dithiobis-succinimidyl propionate SAM layer, and ethanolamine was used for surface-blocking. The charge transfer resistance (R_ct_) was observed to decrease with increasing TNFα concentration, which could be detected up to a concentration of 57 fM [132].

Sharma et al. developed an impedimetric immunosensor for IL-8 detection using a recombinant antibody-mimetic protein as the biorecognition element, which was selected by Phage display, and expressed using a pET11 vector. IL-8 (or CXCL8) is a chemokine assigned with the task of recruiting neutrophils chemotactically at the site of injury [133]. This sensor consisted of Au electrodes deposited on a silicon substrate functionalized with a monothiol-alkane-PEG-acid capacitative SAM for Ab attachment. The impedance phase shift was monitored in response to samples containing varying concentrations of IL-8, which could be detected at concentrations as low as 90 fg/mL [134].

Russel et al. demonstrated a unique microelectrode-based immunosensor for the real-time detection of IL-6, comprising an array of eight Au discs, with needle-shaped microelectrodes on a silicon substrate. Anti-IL-6 Ab was covalently immobilized on Au discs. Both DPV and EIS were employed for real-time detection of IL-6, using potassium ferricyanide/ferrocyanide (K_3_[Fe(CN)_6_]/K_4_[Fe(CN)_6_]) as the redox couple. Interestingly, the authors observed a decrease in impedance and increase in peak current upon antigen binding, which contradicts the typical sensor response for macroelectrodes. This could likely be due to the exposure of channels through the densely packed SAM layer on the microelectrode surface upon Agn-Ab binding, rendering better accessibility to the redox couple. Furthermore, microelectrodes offer advantages of reduced IR_s_ drop and enhanced signal-to-noise ratio. Grafting of additional conductive carbon-based or nanoparticle layers is also not required [135].

Deng et al. developed a unique sandwich type immunosensor based on post-processing of nanolabels for enhanced signal generation. The working principle of this immunosensor relied on the growth and positively charged-capping of AuNPs, which were conjugated to anti-IL-6 Ab_2_ for signal generation. The detection signal is the redox current generated at the electrode by K_3_[Fe(CN)_6_]/K_4_[Fe(CN)_6_] pair. In the absence of the IL-6-AuNP-Ab_2_ immunocomplex, the densely packed SAM layer of 11-mercaptoundecanoic acid prevents [Fe(CN)_6_]^4−/3−^ from reaching the Au electrode, thus resulting in no current generation. IL-6 mediated immobilization of AuNP-Ab_2_, followed by growth and positively charged-capping with cetyltrimethylammonium bromide (CTAB) led to the formation of tunnels in the SAM layer. This gives the redox couple a better chance of undergoing redox reaction at the electrode surface [136].

A summary of design and sensor preparation process as well as sensor performance parameters are presented on Table 5 and Table 6.

**Table 5:** SAM-enabled impedimetric immunosensors: Design and sensor preparation.

Sl. no. Ref.	Transducer Components	Redox Species	Bio-Recognition Element	Blocking Agent	Preparation and Immobilization (Brief)
1. [125]	Si/SiO_2_/SWCNT/AuNP/Ab	[Fe(CN)_6_]^4−/3−^	Anti-IL-6 Ab	BSA	Si/SiO_2_/SWCNT: prepared by ethanol chemical vapor condensation on Si/SiO_2_ wafer substrate Si/SiO_2_/SWCNT+ HAuCl_4_: AuNP formation by electrochemical deposition Si/SiO_2_/SWCNT/AuNP+ mercaptoacetic acid+ Ab: Au-S covalent bonding, amide bond formation with EDC/NHS catalyst
2. [126]	Au/CMA/Ab	[Fe(CN)_6_]^4−/3−^	Anti-TNFα Ab	Ethanolamine	Au microelectrodes+ CMA: Diazotization of CMA in presence of NaNO_3_, HCl; electrodeposition on Au by CV Au/CMA+ Ab: covalent immobilization; amide bond formation with EDC/NHS catalyst
3. [96]	ITO/P3/Ab	[Fe(CN)_6_]^4−/3−^	Anti-TNFα Ab	BSA	ITO(-OH gr.) + P3(-COOH gr.): ester bond formation: SAM formation ITO/P3+ Ab: amide bond formation with EDC/NHS catalyst
4. [127]	Au/NAC/Ab	-	Anti-IFNγ Ab	Ethanolamine	Au electrode+ acetylcysteine: SAM formation by Au-S chemistry Au/NAC+ Ab: amide bond formation with EDC/NHS catalyst
5. [128]	Au/PEG/anti-TGF-β1 Ab, anti-HA Ab	[Fe(CN)_6_]^4−/3−^	Anti-TGF-β1 Ab	-	Cr-Au layered interdigited electrodes prepared with lithography + etching Au electrode+ COOH-PEG-SH: Au-S chemistry; SAM formation Au/PEG+ Ab: amide bond formation with EDC/NHS catalyst
6. [130]	PI/Au-Ab	[Fe(CN)_6_]^4−/3−^	Anti-IL-10 Ab	-	Au layered on PI by soft lithography + etching Carboxyl diazonium reduced with CV, grafted on PI/Au electrode Au-COOH+ Ab: amide bond formation with EDC/NHS catalyst
7. [131]	Si/HfO_2_/TESUD/Ab	-	Anti-IL-10 Ab	MeO-PEG-NH_2_ in triethylamine	HfO_2_ grown on Si substrate by atomic layer deposition Si/HfO_2_(-OH gr.) + TESUD: chemical vapor deposition, SAM formation PDMS stamp + Ab: physisorption Si/HfO_2_/TESUD(-CHO gr.) + PDMS/Ab(-NH_2_ gr.): microcontact printing; imine bond formation
8. [132]	Si/Au/DSP/Ab/EA	[Fe(CN)_6_]^4−/3−^	Anti-TNFα Ab	Ethanolamine	Si/Ti/Au microelectrode arrays preparation: photolithography, etching Si/Au + DSP: DSP reduced with TCEP; immobilization with Au-S chemistry Si/Au/DSP + Ab: Amide bond formation Si/Au/DSP/Ab + EA: Amide bond formation
9. [134]	Si/SiO_2_/Ti-Au/mcp	-	Anti-IL-8 mcp	Ethanolamine	Phage display selection of IL-8 binding Ab-mimetic capture protein mcp coding region sub-cloned in pET11 vector; expressed recombinant mcp purified Si/SiO_2_ layered with Ti-Au Au+ SH-(CH_2_)_11_-(OCH_2_CH_2_)_6_-OCH_2_-COOH (monothiol-alkane-PEG-acid): SAM formation with Au-S chemistry Au/SH-(CH_2_)_11_-(OCH_2_CH_2_)_6_-OCH_2_-COOH + mcp(-NH_2_): amide bond formation with EDC/NHS catalyst
10. [135]	Si/SiO_2_/Ti:Au disc/Ab needle-shaped microelectrode	[Fe(CN)_6_]^4−/3−^	Anti-IL-6 Ab	6-mercapto-1-hexanol	Si/SiO_2_/Ti:Au disc: lift-off lithography; reactive ion etching Ab + sulfo-LC-SPDP: amide bond formation Ab/sulfo-LC-SPDP + DTT + Au disc: Au-S chemistry
11. [136]	AuNP-Ab_2_ Au/MUA/Ab_1_ Growth solution: HAuCl_4_ + ascorbic acid+ CTAB	[Fe(CN)_6_]^4−/3−^	Anti-IL-6 Ab_1_, Ab_2_	BSA	AuNP: citrate reduction of HAuCl_4_ AuNP + Ab_2_: adsorption Au electrode + 11-MUA: Au-S chemistry Au/MUA+ Ab_1_: amide bond formation with EDC/NHS catalyst HAuCl_4_ + ascorbic acid + CTAB: growth of immobilized AuNP-Ab_1_ by reduction of HAuCl_4_ upon AuNP seeds; positively charged capping of AuNPs

Common abbreviations: CMA = 4-carboxymethylaniline, AuNP = Gold nanoparticles, CNT = Carbon nanotube, SWCNT = single-walled CNT, ITO = Indium tin oxide, P3 = poly(3-thiophene acetic acid), NAC = N-acetylcysteine, Me = methyl group, PEG = polyethylene glycol, HA = Hyaluronic acid, PI = Polyimide, TESUD = (11-triethoxysilyl)undecanal, DSP = Dithiobis-succinimidyl propionate, EA = ethanolamine, mcp = recombinant Ab-mimetic capture protein, MUA = 11-mercaptoundecanoic acid, CTAB = cetyltrimethylammonium bromide; Ab_1_ = capture antibody, Ab_2_ = signal antibody; PDMS = polydimethylsiloxane, EDC = 1-ethyl-3-(3-dimethylaminopropyl)carbodiimide, NHS = N-hydroxysuccinimide, Sulfo-LC-SPDP = Sulfosuccinimidyl 6-[3’-(2-pyridyl dithio) propionamido] hexanoate, DTT = Dithiothreitol, TCEP = tris(2-carboxyethyl) phosphine hydrochloride.

**Table 6:** SAM-enabled impedimetric immunosensors: Specifications and performance characteristics.

Sl. no. [Ref.]	Detection Technique	Limit of Detection	Range of Detection	Interfering Species Tested	Incubation Time	Sample Type	Reproducibility	Stability	Repeatability
1. [125]	EIS	0.01 fg/mL	0.01–100 fg/mL	Serum, glucose, cysteine, Epinephrine	-	IL-6 in buffer Spiked serum	-	After 1 month- initial R_ct_ retained	-
2. [126]	EIS	-	1–15 pg/mL	rhIL-8 rhIL-1	+TNFα/30 min	rTNFα in PBS Spiked artificial saliva Real human saliva sample	-	-	No significant change in signal after 3 subsequent detections
3. [96]	EIS	3.7 fg/mL	0.01–2 pg/mL	Drugs- ampicillin, amoxicillin, erythromycin, clarithromycin, acetylsalicylic acid; Proteins- biotin, albumin; Biomarkers- SOX2, MAGE1, RACK1, HER2, VEGFR	45 min	TNFα in PBS Human saliva sample Human serum sample	80 electrodes used to draw 10 calibration plots; RSD of slopes = 2.5%	After 8 weeks, impedance decreased to 50% of initial value	Good response for 6 cycles
4. [127]	EIS CA	0.02 fg/mL	0–12 pg/mL	IL-2	-	rIFNγ in PBS	-	-	Removal of non-specifically adsorbed proteins/KCl solution, regeneration with: SAM wipeout/thioctic acid/potential pulses+ SAM reassembly; 10% repeatability
5. [128]	EIS	0.57 ng/mL	1–1000 ng/mL	BSA	+TGF-β1, HA/30 min	TGF-β1 in PBS Serum sample	-	-	-
6. [130]	EIS	-	1–15 pg/mL	-	-	IL-10 in buffer	-	-	-
7. [131]	EIS	0.1 pg/mL	0.1–20 pg/mL	TNFα, IL-1β	+IL-10/1 h	rh1L-10 in PBS	-	-	-
8. [132]	EIS	~57 fM	1–100 pg/mL	IFNγ	+TNFα/15 min	TNFα in culture media	-	-	-
9. [134]	EIS	90 fg/mL	0.0009–900 ng/mL	BSA	+IL-8/15 min	IL-8 in spiked horse serum	-	-	-
10. [135]	DPV EIS	- -	- 0–60 pg/mL	BSA, Enterotoxin A	+IL-6/2.5 min (real-time detection)	IL-6 in PBS Spiked human serum	-	-	-
11. [136]	SWV	2 pg/mL	0.005–50 ng/mL	hIgG, lysozyme, BSA, AFP	+IL-6/1 h +AuNP-Ab_2_/1 h +growth solution/1h	IL-6 in PBS	(6 assays, at [IL-6] = 10 ng/mL) RSD = 7.9%	-	-

Common abbreviations: PBS = Phosphate-buffered saline, rhIL = Recombinant human interleukin, BSA = Bovine Serum Albumin, hIg = Human immunoglobulin protein, SOX2 = Sex determining region Y-box 2, MAGE1 = Melanoma associated antigen 1, RACK1 = Receptor for activated C kinase 1, HER2 = Human epidermal growth factor receptor, VEGFR = vascular endothelial growth factor receptor, AFP = α-1-fetoprotein.

#### 2.1.4. Heavy Metal Nanoparticle-Labeled Immunosensors

**Figure 11:**
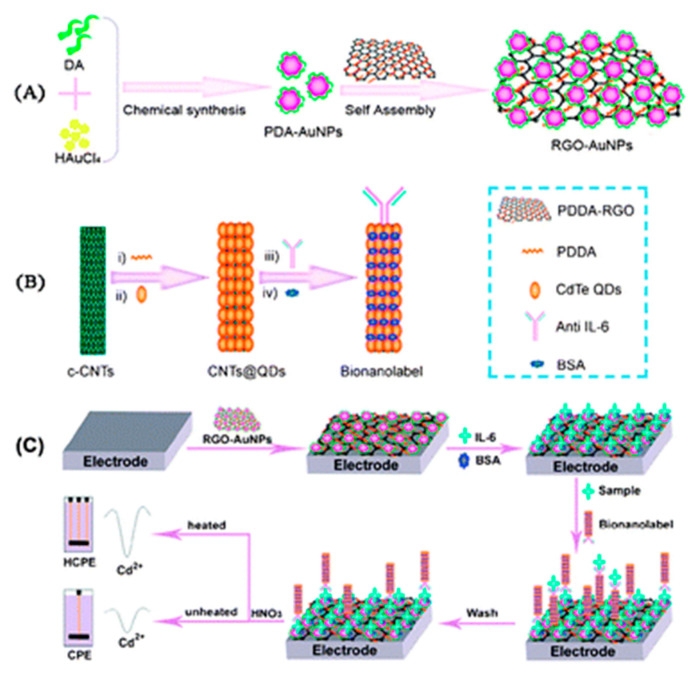
Schematic illustrations of (**A**) the preparation of RGO-AuNPs nanocomposite, (**B**) assembly procedure of CNTs@QDs-anti-IL-6 Ab bionanolabel and (**C**) fabrication and measurement process of the competitive immunosensor. Surface bound IL-6 and sample IL-6 compete for binding with the bionanolabel, and the depletion in the bound Cd^2+^ is measured with stripping voltammetry on a heated SPCE, proportionate to the IL-6 concentration. Reprinted from [137] with permission from The Royal Society of Chemistry.

Non-enzymatic labels have also been employed as reporters for electrochemical immunosensors. Heavy metal nanoparticles are commonly used as non-enzymatic reporters due to their enhanced stability and reproducibility compared with enzymes. Peng et al. reported an IL-6 immunosensor using a unique nanocomposite label consisting of a hollow titanium phosphate (TiP) shell coated with polystyrene, silver nanoparticles (AgNP) and anti-IL-6 Ab_2_. TiP was an ideal choice for dense AgNP deposition, owing to the ion exchange of Ag^+^ with H^+^ present in a large number of –OH groups in TiP. The other component of this sensor was Fe_3_O_4_ nanoparticles functionalized with anti-IL-6 Ab_1_. In the presence of IL-6 Agn, Ab_2_-AgNP-TiP spheres were assembled via an external magnet, through an Ab_2_-Agn-Ab_1_/Fe_3_O_4_ bridge. The captured AgNPs were subsequently dissociated and quantified using stripping voltammetry, and the signal was proportional to the IL-6 concentration [97]. Another unique label was reported by Zhang et al., which comprised of CNTs coated with CdTe quantum dots and a single capture Ab, for IL-6 detection. This sensor also employed a GCE modified with a PDDA-graphene oxide (GO) SAM. Polydopamine (PDA) coated AuNPs and IL-6 (in saturating concentrations) were adsorbed on this layer. Sample IL-6 competed with surface-bound IL-6 for the binding sites of captured Ab. This led to the depletion of CNT@CdTe QDs-Ab on the sensor surface with increasing sample IL-6 concentration (Figure 11). Voltammetric measurements were performed on a separate heated carbon paste electrode, and Cd^+^ stripping gave off the oxidation current used for the corresponding IL-6 quantification. A heated electrode was found to enhance the stripping kinetics as opposed to a non-heated one [137]. PDA was also utilized by Shi et al. for coating a polystyrene label, which was functionalized with AgNPs and Ab_2_. PDA offers the advantages of self-polymerization, good substrate adhesion and good biocompatibility [138]. Ab_1_ was attached to graphene nanoribbons (GNR) stacked on a heated SPCE. Ag^+^ stripping was performed for voltammetric measurements of IL-6, similar to the previous study [139]. Silica nanoparticles (SiNPs) were used as a unique label in the study reported by Yuan et al. Two constructs were used for the detection of TNFα. The first consisted of SiNPs coated with fibrous poly(glycidyl methacrylate) (PGMA) chains, grown with surface-initiated atom transfer radical polymerization (SI-ATRP). CdTe quantum dots were covalently embedded onto this layer alongside Ab_2_. The second construct consisted of a poly(abscisic acid) coated Au substrate, loaded with Ab_1_. The SiNPs exhibited a smooth surface, enabling uniform and consistent molecular adsorption. Cd^2+^ ions were electrodeposited, followed by stripping on a bismuth film layered GCE for generating the detection signal [140]. Anodic stripping of Cd^+^ was also used by Wang et al. for IFNγ detection. Magnetic nanoparticles (MNPs) coated with polyaniline (PANI) and Ab_1_ were used to magnetically capture IFNγ from the sample. This step was followed by the attachment of CdS NPs via Ab_2_ binding, which were conjugated to AuNPs. Upon dissolution of Cd^2+^ on a SPCE with HNO_3_ solution, Cd^2+^ was quantified with anodic stripping voltammetry [141].

A summary of design and sensor preparation process as well as sensor performance parameters are presented on Table 7 and Table 8.

**Table 7:** Heavy metal nanoparticle-labeled immunosensors: Design and sensor preparation.

Sl. no. Ref.	Transducer Components	Redox Species	Bio-Recognition Element	Blocking Agent	Preparation and Immobilization (Brief)
1. [97]	Ab_2_-AgNP-TiP Fe_3_O_4_-Ab_1_	Ag^0/1+^	Anti-IL-6 Ab_1_, Ab_2_	BSA	AgNP + TiP: ion exchange AgNP-TiP + Ab_2_: covalent bond using mercapto or primary amino group of Ab_2_ Fe_3_O_4_ + Ab_1_: assembled with external magnet
2. [137]	GCE/PDDA-RGO-AuNP-PDA/IL-6/BSA PDDA-CNT@CdTe QDs-Ab/BSA HCPE (working electrode)	Cd^1+/2+^	Anti-IL-6 Ab	BSA	AuNP-PDA: by reduction of HAuCl_4_, oxidation and polymerization of dopamine (DA) PDDA-RGO + AuNP-PDA: Adsorption GCE/PDDA-RGO-AuNP-PDA + IL-6: Adsorption MPA capped CdTe QDs: prepared from MPA, CdCl_2_ & NaHTe CNT(-COOH gr.) + PDDA: covalent bonding PDDA-CNT + CdTe QDs (mercaptopropanoic acid capped): Adsorption PDDA-CNT@CdTe QDs + anti-IL-6 Ab: amide bond formation with EDC catalyst Competitive binding of Ab with IL-6 in sample vs. IL-6 in modified GCE; bound CdTe dissolved with HNO_3_, Cd^2 +^ deposited, followed by stripping at HCPE; HCPE heated with high frequency AC at the deposition step
3. [139]	Ab_2_/PS@PDA/AgNP HSPCE/GNR/Ab_1_	Ag^0/1+^	Anti-IL-6 Ab_1_, Ab_2_	-	PS + DA (dopamine): Self polymerization of DA; adhesion of PDA to PS GNRs: prepared from GONRs (graphene oxide nanoribbons) reduced with N_2_H_4_ PS@PDA/AgNP: prepared from AgNO_3_ PS@PDA/AgNP + Ab_2_: amide bond formation with EDC/NHS catalyst HSPCE/GNR + Ab_1_: Adsorption; HSPCE heated with high frequency AC
4. [140]	SiNP/PGMA/CdTe QD/Ab_2_ Au-PAB-Ab_1_ BFE	Cd^1+/2+^	Anti-TNFα Ab_1_, Ab_2_	BSA	SiO_2_ NP + APTES/toluene: SiO_2_-NH_2_ NP formation SiO_2_-NH_2_ NP + trimethylamine/toluene/BriBuBr: SiO_2_-Br NP formation SiO_2_-Br NP + dimethylformamide + GMA + CuBr: surface initiated atom transfer radical polymerization; SiNP/PGMA formation CdTe-S-CH_2_COOH + SiNP/PGMA (-(CH)_2_O gr.): ring-open reaction SiNP/PGMA/CdTe QD + Ab_2_: covalent immobilization with EDC/NHS catalyst
5. [141]	MNP/PANI/Ab_1_ AuNP-Ab_2_-CdS NP SPCE	Cd^1+/2+^	Anti-IFNγ Ab_1_, Ab_2_	Casein/tris buffer for AuNP BSA for MNP	MNP + PANI: coating MNP/PANI + Ab_1_: adsorption AuNP + Ab_2_: adsorption CdCl_2_ + SHCH_2_COOH + Na_2_S → CdS NP capped with (-SCH_2_COOH) AuNP-Ab_2_ + 3’ SH-poly[A]-NH_2_ 5’ linker + CdS NP: linkage with EDC/NHS catalyst; Au-S chemistry MNP-IFNγ separated magnetically; resuspended in HNO_3_ + Bi/acetate buffer

Common abbreviations: TiP = Titanium phosphate hollow spheres, PDDA = poly(diallyldimethylammonium chloride), RGO = graphene oxide, PDA = polydopamine, CdTe QDs = Cadmium telluride quantum dots, HCPE = heated carbon paste electrode, PS = Polystyrene, GNR = Graphene nanoribbons, SiNP = silica nanoparticles, PGMA = poly(glycidyl methacrylate), PAB = poly(abscisic acid), MNP = magnetic nanoparticles, PANI = polyaniline, CNT = Carbon nanotube, GCE = Glassy carbon electrode, BFE = Bismuth film modified GCE, AuNP = Gold nanoparticles, AgNP = Silver nanoparticles, SPCE = Screen printed carbon electrode, HSPCE = Heated SPCE; Ab_1_ = capture antibody, Ab_2_ = signal antibody; EDC = 1-ethyl-3-(3-dimethylaminopropyl)carbodiimide, NHS = N-hydroxysuccinimide.

**Table 8:** Heavy metal nanoparticle-labeled immunosensors: Specifications and performance characteristics.

Sl. no. Ref.	Detection Technique	Limit of Detection	Range of Detection	Interfering Species Tested	Incubation Time	Sample Type	Reproducibility	Stability	Repeatability
1. [97]	Electrodeposition followed by stripping with DPV	0.1 pg/mL	0.0005–10 ng/mL	TNF-α, hIgG, CEA	+IL-6/40 min +Ab_2_-AgNP-TiP/40 min	IL-6 in PBS Spiked serum	(2 assays) RSD = 8.0%	After 2 months- 94.2% of initial signal retained	-
2. [137]	ASV	0.033 pg/mL for HCPE	0.1–100 pg/mL for HCPE	CEA, CRP, TNF-α, BSA	+IL-6, +CNT@CdTe QDs-Ab/40 min +dissolution of Cd^2 +^ with HNO_3_/30 min	IL-6 in buffer Spiked serum	(5 assays, at [IL-6] = 10 pg/mL) RSD = 4.1%	After 1 week- >90% of initial signal retained	-
3. [139]	Stripping SWV	0.1 pg/mL	0.001–1000 ng/mL	BSA, CEA, CTnI, IgG	+IL-6/50 min +Ab_2_/PS@PDA/AgNP/50 min	IL-6 in buffer Spiked serum	(5 assays) initial signal significantly retained at [IL-6] = 0.1 ng/mL	After 2 weeks- initial signal not significantly changed	-
4. [140]	SWV	3.0 pg/mL	0.01–100 ng/mL	-	AuNP-PAB-Ab_1_ + TNFα/45 min +SiNP/PGMA/CdTe QD/Ab_2_/45 min	TNFα in buffer	Intra-assay precision (4 runs) CV=5.1% Inter-assay precision (4 assays) CV = 6.7%	After 2 weeks, no apparent change in signal	After 6 cycles & regeneration/glycine-HCl, 95.7% of initial signal retained
5. [141]	SWASV	0.4 pg/mL	0.01–1 IU/mL	-	IFNγ + MNP/PANI/Ab_1_/20 min +BSA blocking/5 min +AuNP-Ab_2_-CdS NP/20 min +HNO_3_/10 min +Bi electrodeposition/10 min	IFNγ in PBS	-	-	-

Common abbreviations: PBS = Phosphate-buffered saline, BSA = Bovine Serum Albumin, CEA = Carcinoembryonic antigen, hIg = Human immunoglobulin protein, CRP = C-reactive protein, CTnI = Cardiac Troponin I.

#### 2.1.5. Redox-Labeled Immunosensors

**Figure 12:**
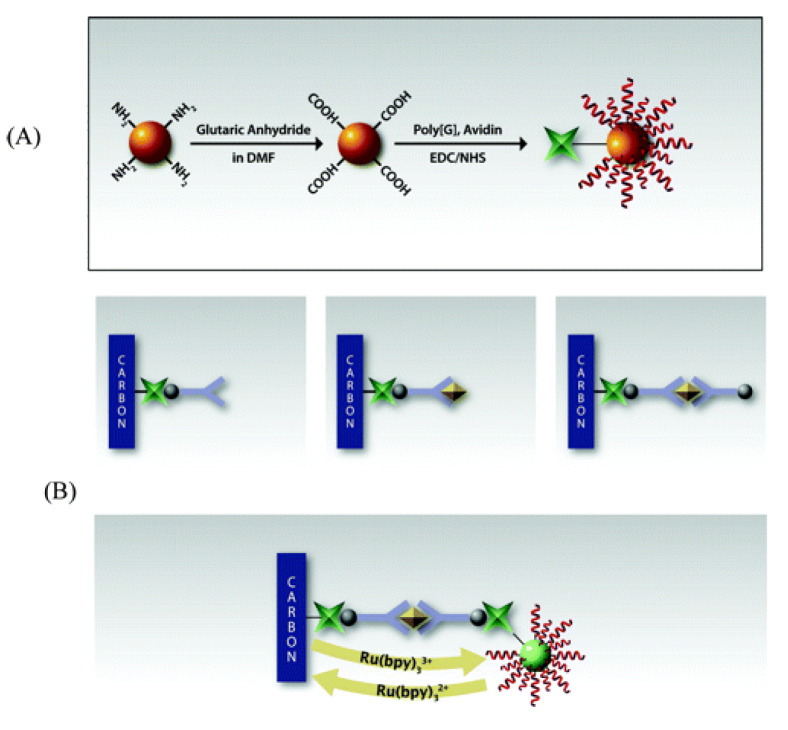
Schematic illustrations of (**A**) the procedure for preparation of poly[G]- and avidin-functionalized SiNP conjugate and (**B**) the procedure and principle for electrochemical immunoassay. Sandwich immunoreaction of TNFα with Ab_1_ and Ab_2_ brings poly[G] close to the electrode, which undergoes oxidation, mediated by Ru(bpy)_3_^2+^. Reprinted from [145] with permission from American Chemical Society.

Redox labels are molecular species that can undergo redox reaction close to an electrode surface to generate an electrochemical signal. By uniquely designing redox labels, the reaction kinetics can be optimized to enhance the sensor performance. Due to the broad range of nanoparticles available, the performance characteristics of the redox labels can be fine-tuned based on the nanoparticle properties, such as electrical conductivity, reaction kinetics, band-gap, etc. A unique nanocomposite label was reported by Li et al. for IL-6 detection, comprising a CaCO_3_ core coated with a porous polyelectrolyte layer. The porous layer consisted of PDDA and poly(styrene sulfonate) (PSS), which was loaded with Fc and Ab_2_. In the presence of IL-6, the redox current of Fc generated an electrochemical signal on an electrode functionalized with GO and Ab_1_ [98]. Redox mediators are often used to enhance the electrochemical signal and detection sensitivity by undergoing coupled redox cycling. Li et al. reported an immunosensor for TNFα detection utilizing two redox mediators, Fe^2+/3+^ of Prussian blue nanoparticles (PB) and Ce^3+/4+^ of a CeO_2_ coating, which served as an artificial peroxidase for generating current from H_2_O_2_ reduction. Anti-TNFα Ab_1_ was immobilized on a GCE modified with AuNP-CNT complexes, and anti-TNFα Ab_2_ was immobilized onto chitosan-coated CeO_2_/PB. PB has shown to be an excellent choice as an electron transfer mediator, when coupled with H_2_O_2_ reduction [142]. Weng et al. reported an immunosensor for TNFα detection that utilized Fe^2+/3+^ as a redox label for signal generation. This sensor consisted of a GCE with K_3_[Fe(CN)_6_] entrapped within a chitosan (CS) layer. CS was cross-linked with glutaraldehyde, and the entire assembly was secured in place with a Nafion coating. The Nafion coating also facilitated the adsorption of the captured Ab. When subject to cyclic voltammetry, [Fe(CN)_6_]^4−/3−^ undergoes a redox reaction, and its anodic current decreased with an increasing TNFα concentration because of the barrier in charge transfer posed by the non-conducting TNFα-anti-TNFα Ab pairs [143]. In another immunosensor proposed by Wang et al., ZnO NPs, loaded with Ab and sealed with a Nafion coating, were used for impedimetric detection of IFNγ on a GCE. [Fe(CN)_6_]^4−/3−^ served as the redox species [144]. Wang et al. prepared a redox label with SiNPs coated with poly(guanine) and avidin (Av), which attracted Bt-Ab_2_. Bt-Av affinity was used to attract Ab_1_ to the Av-functionalized SPCE (Figure 12). When subjected to a potential sweep in the positive range, electroactive guanine was irreversibly oxidized, and quickly exhausted in the vicinity of the electrode surface. To prolong this effect, a mediator such as Ru(bpy)_3_^2+^ was used to act as an electron messenger. The transient nature of the mediator ensured its effectivity even in very low concentrations. This immunosensor was used for measurements of TNFα [145].

A summary of design and sensor preparation process as well as sensor performance parameters are presented on Table 9 and Table 10.

**Table 9:** Redox-labeled immunosensors: Design and sensor preparation.

Sl. no. Ref.	Transducer Components	Redox Species	Bio-Recognition Element	Blocking Agent	Preparation and Immobilization (Brief)
1. [98]	GCE/GO-Ab_1_ CaCO_3_/Fc-PPN-Ab_2_	Fe^2+/3 +^ of Fc	Anti-IL-6 Ab_1_, Ab_2_	BSA	CaCO_3_ + PDDA + PSS + Fc: Adsorption CaCO_3_/Fc-PPN + Ab_2_: Electrostatic Adsorption GCE/GO + Ab_1_: Amide bond formation with EDC/NHS catalyst
2. [142]	CeO_2_/CS-PB-Ab_2_ GCE/CNT/PDDA/AuNP/Ab_1_	PB (Fe^2+/2+^/Fe^2+/3+^/Fe^3+/3+^) Ce^3+/4+^ H_2_O_2_/H_2_O	Anti-TNFα Ab_1_, Ab_2_	BSA	CeO_2_ NP/CS (+ve) + Fe(CN)_6_^3−^: Adsorption CeO_2_/CS/Fe(CN)_6_^3−^ + FeCl_2_: PB formation CeO_2_/CS-PB + Ab_2_ + glutaraldehyde: imine bond formation; CS-Ab_2_ cross-linking AuNP: citrate reduction of HAuCl_4_ CNT(-COOH gr.) + PDDA(+ve) + AuNP(-ve) + Ab_1_: Adsorption
3. [143]	GCE/K-CS-GA/Ab	[Fe(CN)_6_]^4−/3−^	Anti-TNFα Ab	BSA	CS (-NH_2_ gr.) + GA (-CHO gr.) + K_3_[Fe(CN)_6_]: crosslinking with imine bond formation; doping with K_3_[Fe(CN)_6_] N + Ab: physical or electrostatic adsorption
4. [144]	GCE/ZnO NP/Ab	[Fe(CN)_6_]^4−/3−^	Anti-IFNγ Ab	BSA	Zn(NO_3_)_2_ + H_2_N_2_ → ZnO NP ZnO NP + Ab: Adsorption ZnO NP/Ab + GCE: drop-casting GCE/ZnO NP/Ab + Nafion: top-coating
5. [145]	SPCE/Av/Bt-Ab_1_ Bt-Ab_2_ SiNP/poly[G]/Av	Guanine (irreversible oxidation) Tris(bipyridine)ruthenium(II) chloride or Ru(bpy)_3_^2+/3+^	Anti-TNFα Ab_1_, Ab_2_	BSA	SiNP(-NH_2_ gr.) + glutaric anhydride + dimethylformamide: SiNP(-COOH gr.) SiNP(-COOH gr.) + Av, poly[G]: covalent immobilization with EDC/NHS catalyst Bt-Ab_1_ + Av/SPCE: affinity bonding

Common abbreviations: PPN = Porous polyelectrolyte nanoparticles composed of poly(diallyldimethylammonium chloride (PDDA) and Poly(styrene sulfonate) (PSS), Fc = Ferrocene, GO = Graphene oxide, PB = Prussian blue nanoparticles, K = K_4_[Fe(CN)_6_], CS = Chitosan, GA = Glutaraldehyde, N = Nafion, SiNP = silica nanoparticles, G = guanine nucleotide, Bt = Biotin, Av = Avidin, poly[G] = 5’ NH_2_-(CH_2_)_12_-(G)_10/20_ 3’, SPCE = Screen printed carbon electrode, GCE = Glassy carbon electrode, CNT = Carbon nanotubes, AuNP = Gold nanoparticles; Ab_1_ = capture antibody, Ab_2_ = signal antibody; EDC = 1-ethyl-3-(3-dimethylaminopropyl)carbodiimide, NHS = N-hydroxysuccinimide.

**Table 10:** Redox-labeled immunosensors: Specifications and performance characteristics.

Sl. no. Ref.	Detection Technique	Limit of Detection	Range of Detection	Interfering Species Tested	Incubation Time	Sample Type	Reproducibility	Stability	Repeatability
1. [98]	SWV	1 pg/mL	0.002–20 ng/mL	hIgG, Lysozyme, AFP, PSA	+IL-6/1 h +CaCO_3_/FC-PPN-Ab_2_/1 h	IL-6 in PBS Spiked serum	(6 assays) RSD = 5.8%	After 30 days- 90% of initial signal retained	92.3% of initial signal retained after 6 regeneration cycles
2. [142]	CA	2 pg/mL	0.005–5 ng/mL	PSA, AFP, CEA	+TNFα/1 h +CeO_2_/CS-PB-Ab_2_/1 h	TNFα in buffer	Intra-assay precision (5 runs, at [TNFα] = 1 ng/mL) RSD = 4.6% Inter-assay precision (5 assays, at [TNFα] = 1 ng/mL) RSD = 7.5%	After 2 weeks, negligible current variation	-
3. [143]	CV	10 pg/mL	0.02–34 ng/mL	CA-125, CA-153, CA 19–9, AFP	+TNFα/1 h	TNFα in PBS Human serum sample	(5 assays, at [TNFα] = 1 ng/mL) RSD = 3.2%	-	-
4. [144]	EIS	0.12 pg/mL	0.0001–0.1 ng/mL	-	+IFNγ/80 min	rIFNγ in PBS Spiked bovine serum	Intra-assay precision (6 runs, at [IFNγ] = 0.01ng/mL) CV = 4.1% Inter-assay precision (6 assays, at [IFNγ] = 0.01ng/mL) CV = 3.9%	After 60 days, no apparent change in R_ct_	-
5. [145]	SWV	2 pM	-	-	+TNFα/45 min + Bt-Ab_2_/45 min +SiNP/poly[G]/Av/45 min	TNFα in PBS	(6 runs, at [TNFα] = 1 ng/mL) RSD = 9.8%	-	-

Common abbreviations: PBS = Phosphate-buffered saline, BSA = Bovine Serum Albumin, CEA = Carcinoembryonic antigen, hIg = Human immunoglobulin protein, AFP = α-1-fetoprotein, PSA = Prostate specific antigen, CA-125 (or -153, 19–9) = Cancer antigen-125.

#### 2.1.6. Magnetoimmunosensors

**Figure 13:**
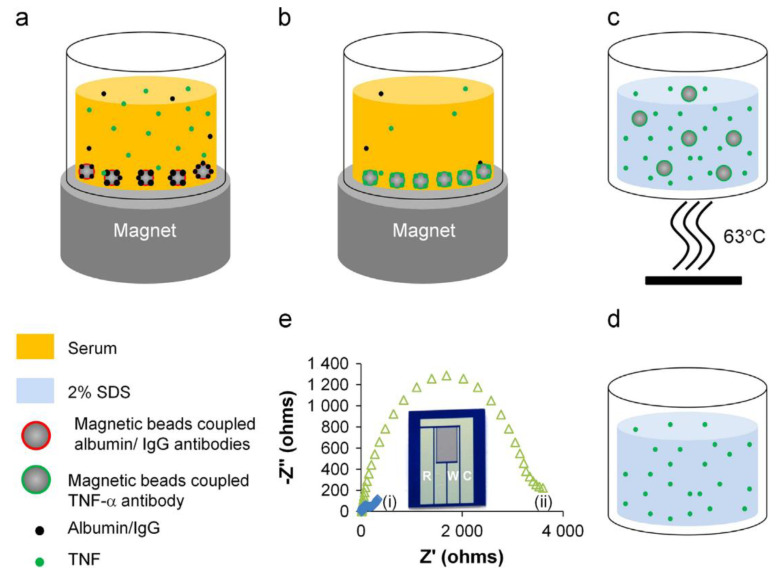
Schematic illustration of a magnetoimmunosensor for TNFα detection (**a**) Serum background is removed using magnetic beads coated with albumin and IgG antibodies. (**b**) The background removed sample is transferred to a new tube, and TNFα is then captured using magnetic bead coated with TNFα antibody. (**c**) The captured TNFα is eluted from the magnetic beads using 2% SDS at 63℃, and (**d**) the magnetic beads are separated out of the eluted sample. (**e**) Impedance spectra of the eluted sample are then analyzed using an EIS technique in which the CSGM electrode is employed as a platform for TNFα adsorption and for EIS analysis. R, W and C labeled on CSGM electrodes denote reference, working and counter electrodes, respectively. Nyquist plots represent impedance spectra recorded from (i) blank chip and (ii) eluted sample of 1pg/ml TNFα spiked in human serum. Reprinted from [99] with permission from Elsevier.

Magnetoimmunosensors utilize magnetic beads (Mbs) to facilitate protein purification, Agn-Ab binding and the transport of Agn-Ab immunocomplexes to the electrode surface using a magnet. Kongsuphol et al. developed a magnetoimmunosensor for the impedimetric detection of TNFα in undiluted serum using magnetic beads coated with anti-TNFα Ab. This sensing strategy involved three main steps. First, the undiluted serum sample was incubated with Mbs coated with anti-albumin and anti-IgG Abs, and the Mb-immunocomplexes were separated using a magnetic rack in order to remove albumin and IgG, which account for a major percentage of background interference. Second, the sample was incubated with Mbs coated with anti-TNFα Ab, and the Mb-immunocomplexes were separated with a magnetic rack to isolate TNFα. Third, TNFα was eluted from Mbs via SDS denaturation, followed by adsorption and impedimetric quantification on a comb structured gold microelectrode (CSGM) (Figure 13). One advantage of this approach is that the specific removal of albumin and IgG in the serum sample prior to TNFα detection greatly minimizes the likelihood of a false negative result [99]. Mbs were used by Eletxigerra et al. for the preparation of a sandwich type amperometric immunosensor for TNFα detection. For this sensor, a HRP (enzyme)-hydroquinone, H_2_O_2_ (substrates) system was used for signal generation on a SPCE. Anti-TNFα Ab_1_ was immobilized on Mbs, and Ab_2_-Bt-SAv-HRP was used as an enzymatic label [146]. Another magnetoimmunosensor developed by Bettazzi et al. employed a unique approach for the immobilization of anti-TNFα Ab_1_ on Mb coated with Protein G, which is known to bear affinity for the F_c_ region of IgG of many species. Ab_1_/Protein G/Mbs were transported to a screen printed graphite electrode (SPGE) using a surface magnet, and upon TNFα binding, formed a sandwich immunocomplex with Ab_2_-Bt-SAv-ALP. An ALP (enzyme)-1-naphthyl phosphate/1-naphthol (substrate) system was used for signal generation. This immunosensor could detect TNFα down to 0.044 ng/mL [147].

A summary of design and sensor preparation process as well as sensor performance parameters are presented on Table 11 and Table 12.

**Table 11:** Magnetoimmunosensors: Design and sensor preparation.

Sl. no. [Ref.]	Transducer Components	Redox Species	Bio-Recognition Element	Blocking Agent	Preparation and Immobilization (Brief)
1. [99]	Mb/anti-albumin Ab Mb/anti-IgG Ab Mb/anti-TNFα Ab Si/SiO_2_/CSGM	[Fe(CN)_6_]^4−/3−^	Anti-TNFα Ab	-	Mb(-COOH gr.) + anti-(albumin/IgG/TNFα) Ab: amide bond formation with EDC/NHS catalyst Si/SiO_2_/CSGM preparation: photolithography TNFα elution with SDS in Tris
2. [146]	Mb/Ab_1_ Bt-Ab_2_ SAv-HRP SPCE	Hydroquinone/Benzoquinone H_2_O_2_/H_2_O	Anti-TNFα Ab_1_, Ab_2_	Ethanolamine	Mb(-COOH gr.) + Ab_1_, Mb(-COOH gr.) + ethanolamine, Ab_2_ + Bt, SAv + HRP : amide bond formation with EDC/NHS Mb/Ab_1_: magnetically captured onto SPCE
3. [147]	SPGE (with magnetic bars) Mb/Protein G/Ab_1_ Bt-Ab_2_ SAv-ALP	1-naphthol (1-NP) (red → ox)	Anti-TNFα Ab_1_, Ab_2_	Rabbit IgG	Mb/Protein G + Ab_1_: affinity bonding Bt-Ab_2_ + SAv-ALP: affinity bonding Mb captured onto SPGE with magnetic bars 1-naphthyl phosphate (1-NPP) converted to 1-naphthol by ALP

Common abbreviations: CSGM = Comb structured Au microelectrode, SPCE = Screen printed carbon electrode, SPGE = Screen printed graphite electrode, Mb = magnetic bead, Bt = Biotin, SAv = Streptavidin, HRP = Horseradish peroxidase, ALP = Alkaline phosphatase; Ab_1_ = capture antibody, Ab_2_ = signal antibody; EDC = 1-ethyl-3-(3-dimethylaminopropyl)carbodiimide, NHS = N-hydroxysuccinimide.

**Table 12:** Magnetoimmunosensors: Specifications and performance characteristics.

Sl. no. [Ref.]	Detection Technique	Limit of Detection	Range of Detection	Interfering Species Tested	Incubation Time	Sample Type	Reproducibility	Stability	Repeatability
1. [99]	EIS	1 pg/mL	1–1000 pg/mL	IL-2	+Mb/anti-(albumin & IgG) Ab/1 h +Mb/anti-TNFα Ab/1 h +TNFα elute on CSGM	TNFα in PBS Spiked human serum	-	-	-
2. [146]	CA	2.0 pg/mL (standard solution) 5.8 pg/mL (spiked serum)	-	hIgG, BSA	+TNFα/1 h +Bt-Ab_2_/1 h +SAv-HRP/10 min	rTNFα in PBS-Tween20 Spiked human serum	(5 assays, same day) RSD = 5.7% (10 assays, different days) RSD = 7.5%	After 2 weeks, no significant decrease in S/N (signal/noise) ratio	-
3. [147]	DPV	0.044 ng/mL	-	-	Mb +TNFα +Bt-Ab_2_/2 h +SAv-Ab_1_/20 min +pNPP/5 min	TNFα in PBS-Tween20	In array (8 assays) RSD = 4%; Batch-to-batch (24 assays) RSD = 6%	-	(disposable)

Common abbreviations: PBS = Phosphate-buffered saline, BSA = Bovine Serum Albumin, hIg = Human immunoglobulin protein.

#### 2.1.7. Flow-Injection/Microfluidic Immunosensors

**Figure 14:**
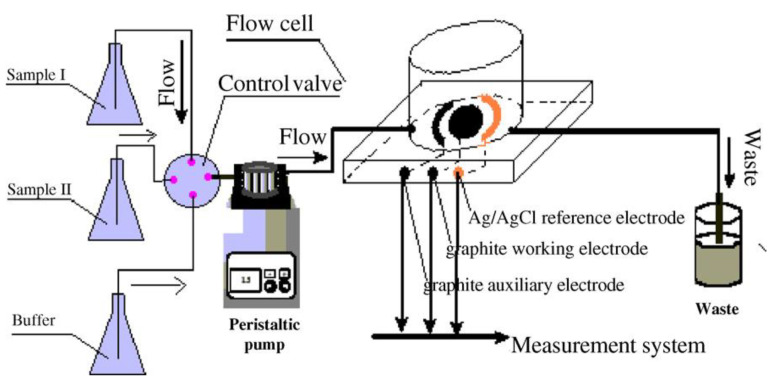
Schematic illustration of the flow-injection immunoassay system for IL-6 determination. HRP-labeled anti-IL-6 Ab immobilized on a SPCE coated with AuNPs generate redox signal with thionine and H_2_O_2_ substrates. This coupled reaction is hindered with IL-6 binding. Reprinted from [101] with permission from Elsevier.

While all of the electrochemical biosensors discussed above are capable of quantitative cytokine detection, most require sample processing and/or sample dilution, which increases the assay complexity and detection time. One approach to simplify this process is to combine a flow-injection or microfluidic system with the sensor to facilitate sample loading and processing. One such flow-injection disposable electrochemical immunosensor was developed by Liang and Mu for the detection of IL-6, by utilizing AuNPs and SPGE for enhanced conductivity, Agn-Ab binding specificity and a HRP (enzyme)-thionine, H_2_O_2_ (substrates) catalytic system (Figure 14). The generated signal reduced proportionately with the increase in sample IL-6 concentration. The binding of IL-6 to HRP-Ab hindered the accessibility of the active center of HRP for oxidization [101]. A four-electrode sensor with a flow-injection system was designed by Berggren et al. for potentiostatic capacitance measurements of IL-6. This sensor consisted of an Au WE coated with anti-IL-6 Ab via a 1,4-butanediol diglycidyl ether SAM and two REs: a platinum wire reference, with a drifting potential, which was compared and calibrated against an external Ag/AgCl electrode with a stable potential. The SAM comprised of cysteamine and 1-dodecanethiol and helped reduce electron transfer through SAM tunnels and increased the capacitive component (C_SAM_) of the electrode surface [148]. Further enhancement in the sensor performance can be achieved through the use of a microfluidic system, as demonstrated by Messina et al. This platform consisted of a microfluidic channel containing monoclonal Ab_1_ immobilized on 3-aminopropyl-modified controlled-pore glass (APCPG). The sample solution was flow-injected into the microchannel, followed by the injection and incubation of solutions containing polyclonal Bt-Ab_2_ and SAv-ALP. The end of the microchannel contained an Au electrode, and an anodic current signal was generated with an ALP (enzyme)-p-aminophenyl phosphate (substrate) system [149]. Ruecha et al. developed a microfluidic immunosensor for IFNγ detection utilizing a screen-printed paper electrode (SPPE). The electrode consisted of graphene coated with polyaniline (PANI), enabling the covalent immobilization of anti-IFNγ Ab. One unique advantage of this paper-based sensor is its ability to be mass produced at low costs [150].

A summary of design and sensor preparation process as well as sensor performance parameters are presented on Table 13 and Table 14.

**Table 13:** Flow-injection/microfluidic immunosensors: Design and sensor preparation.

Sl. no. [Ref.]	Transducer Components	Redox Species	Bio-Recognition Element	Blocking Agent	Preparation and Immobilization (Brief)
1. [101]	HRP-Ab-AuNP/BSA/SPGE	Thionine (TH/TH(H^+^)) H_2_O_2_/H_2_O	Anti-IL-6 Ab	BSA	HRP-Ab + AuNP + BSA: entrapment, covalent bonding with glutaraldehyde
2. [148]	Au/cysteamine/BDE/Ab	-	Anti-IL-6 Ab	1-dodecanethiol	Au rods + cysteamine + BDE + Ab: Au-S bond formation; cross-linking of Ab and cysteamine with BDE
3. [149]	APCPG/Ab_1_ Au electrode Bt-Ab_2_ SAv-ALP Carrier stream: PBS with skim milk	p-aminophenol (pAP)/p-benzoquinoneimine (QI)	Anti-IL-6 Ab_1_, Ab_2_	Skim milk	APCPG + glutaraldehyde + Ab_1_: imine bond formation Bt + SAv: Affinity bonding pAP converted from p-aminophenyl phosphate (pAPP)
4. [150]	SPPE/PANI-G/Ab	[Fe(CN)_6_]^4−/3−^	Anti-IFNγ Ab	BSA	Paper (wax-printed) + aniline/H_2_SO_4_: Electropolymerization by CV PANI-G + Ab: amide bond formation with EDC/NHS catalyst

Common abbreviations: SPGE = Screen printed graphite electrode, BDE = 1,4-butanediol diglycidyl ether, APCPG = 3-aminopropyl- modified controlled-pore glass, SPPE = Screen printed paper electrode, PANI = polyaniline, G = graphene; Ab_1_ = capture antibody, Ab_2_ = signal antibody; EDC = 1-ethyl-3-(3-dimethylaminopropyl)carbodiimide, NHS = N-hydroxysuccinimide.

**Table 14:** Flow-injection/microfluidic immunosensors: Specifications and performance characteristics.

Sl. no. [Ref.]	Detection Technique	Limit of Detection	Range of Detection	Interfering Species Tested	Incubation Time	Sample Type	Reproducibility	Stability	Repeatability
1. [101]	DPV	1.0 ng/L	5–100 ng/L	-	+IL-6/45 min +washing, enzymatic reaction & detection/~5 min	IL-6 in acetate buffer IL-6 in serum specimen	Intra-assay precision (5 runs, at [IL-6] = 50 ng/L) CV = 4.7% Inter-assay precision (5 assays, at [IL-6] = 50 ng/L) CV = 5.4%	After 10 days- 77.6% of initial signal retained	(one-time use, disposable biosensor)
2. [148]	Potentiostatic detection	-	0.5 fM–0.5 pM	IL-2	10 min	rIL-6 in PBS/Tween20/NaN_3_	30–40%	-	(sensor cannot be regenerated)
3. [149]	CA	0.41 pg/mL	-	-	25 min = serum sample+ Bt-Ab_2_/5 min +washing/3 min +SAv-ALP/5 min +washing/3 min +pAPP/5 min +detection/2 min	IL-6 standard solution (ELISA kit) IL-6 in human serum sample	Intra-assay precision (5 runs, at [IL-6] = 200 pg/mL) CV = 2.74% Inter-assay precision (5 assays, at [IL-6] = 200 pg/mL) CV = 5.62%	-	sensor could be used for ≥ 100 determinations following regeneration with glycine-HCl desorption buffer+ PBS washing
4. [150]	EIS	3.4 pg/mL	5–1000 pg/mL	BSA	+IFNγ/30 min	rIFNγ in PBS Spiked human serum (proteins precipitated with CCl_3_COOH)	RSD < 5% (5 assays)	After 2 weeks, 94% of initial R_ct_ retained	(disposable)

Common abbreviations: PBS = Phosphate-buffered saline, BSA = Bovine Serum Albumin.

#### 2.1.8. FET-Based Biosensors

**Figure 15:**
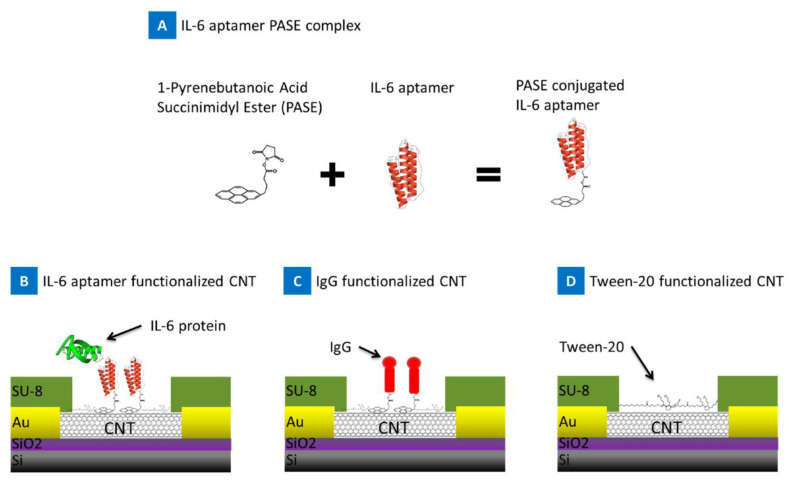
(**A**) Schematic illustration depicting the reaction of 1-pyrenebutanoic acid succinimidyl ester (PASE) with IL-6 Apt forming the IL-6 aptamer PASE complex. Schematic illustrations of the CNT FET sensor functionalized with (**B**) IL-6 Apt PASE complex as positive control, (**C**) IgG as a negative control and (**D**) tween-20 blocking agent as a negative control. Reprinted from [100] with permission from MDPI.

Recently, FET-based biosensors have gained much attention due to their fast response time, miniaturized size and large amplification of potentiometric signal. A liquid gated FET biosensor for IL-6 detection was reported by Huang et al., which employed GO on a silicon substrate for transduction, and anti-IL-6 Ab for biorecognition. The edges of GO were grown using ethanol-chemical vapor deposition for enhanced surface uniformity and higher electrical conductivity [151]. Khosravi et al. designed a FET microarray biosensor for IL-6 detection consisting of a SWCNT/SiO_2_/Si gate coated with RNA Apts. The interaction between IL-6 and RNA Apts caused a change in the conductance proportional to the IL-6 concentration (Figure 15). Using this approach, IL-6 could be detected in real time, with an increase in IL-6 concentration inducing a characteristic spike, followed by a decrease in the sensor conductance [100].

An FET-like aptasensor was devised by Farid et al. for IFNγ detection, which utilized pyrene-terminated Apt stacked on a single layer of graphene (G) on a PDMS substrate. The IFNγ sample on PDMS/G/Apt formed the liquid gate of the FET. A unique feature about this configuration is the small size of the Apt, which ensured that the change in surface charge induced by bound IFNγ does not exceed the Debye length, beyond which charges tend to be masked and ineffective in influencing the gate voltage (V_G_). The parameter used here is current (I_Dirac_) at the Dirac point (voltage of minimum conductance). The right side of Dirac point constitutes the n-conduction region (major charge carriers: electrons), and the left side constitutes the p-conduction region (major charge carriers: holes). Upon IFNγ binding, there was not only an increase in I_Dirac_, but also a shift in the Dirac point, which was successfully correlated with IFNγ concentration in a decaying exponential plot [152].

A summary of design and sensor preparation process as well as sensor performance parameters are presented on Table 15 and Table 16.

**Table 15:** FET-based biosensors: Design and sensor preparation.

Sl. No. [Ref.]	Transducer Components	Redox Species	Bio-Recognition Element	Blocking Agent	Preparation and Immobilization (Brief)
1. [151]	SiO_2_/GO-Ethanol/Ab liquid-gate	-	Anti-IL-6 Ab	BSA Ethanolamine	SiO_2_ + APTES + GO: Silanization, electrostatic attachment SiO_2_/GO + Ethanol: Carbon deposition on the edges of GO flakes by Ethanol chemical vapor deposition SiO_2_/GO-Ethanol + 1-pyrenebutanoic acid, succinimidyl ester + Ab: Amide bond formation, covalent attachment of Ab
2. [100]	Si/SiO_2_/SWCNT/PASE/Apt liquid gate Ni/Au/Si/SiO_2_ source and drain electrodes	-	Anti-IL-6 RNA Apt	-	SWCNT + PASE: pyrene rings adsorption onto sidewalls of nanotube SWCNT/PASE + 5’-NH_2_ modified Apt: amide bond formation CNT + Si/SiO_2_: photolithography, etching
3. [152]	PDMS/G/Apt gate	-	Anti-IFNγ Apt	-	Graphene grown with low pressure chemical vapor deposition Source & drain electrodes placed on Ag terminals; V_G_ applied w.r.t. Ag/AgCl 5’ pyrene-Apt (DNA) + G: π-stacking; immobilization

Common abbreviations: GO = Graphene oxide flakes, SWCNT = Single-wall carbon nanotube, PASE = 1-pyrenebutanoic acid, succinimidyl ester, PDMS = polydimethylsiloxane, G = graphene monolayer; Ab = antibody, Apt = aptamer; APTES = (3-aminopropyl)triethoxysilane.

**Table 16:** FET-based biosensors: Specifications and performance characteristics.

Sl. No. [Ref.]	Detection Technique	Limit of Detection	Range of Detection	Interfering Species Tested	Incubation Time	Sample Type	Reproducibility	Stability	Repeatability
1. [151]	FET based detection	4.7 pg/mL	-	-	-	IL-6 in buffer	-	-	-
2. [100]	FET based detection	1 pg/mL	1–100 pg/mL	BSA, PBS	(real-time detection)	IL-6 in MgCl_2_/PBS Spiked blood	-	-	-
3. [152]	FET based detection	83 pM	~0 nM- ~10 µM	BSA, Papain	-	rIFNγ in PBS	-	-	-

Common abbreviations: PBS = Phosphate-buffered saline, BSA = Bovine Serum Albumin.

#### 2.1.9. Biosensors for Multiplexed Cytokine Detection

While most of the biosensors described above were designed to detect a single cytokine, researchers have also developed sensors capable of detecting multiple cytokines simultaneously. Typically, this is achieved by using two or more WEs which are coated with different biorecognition elements and a shared RE and CE. Alternatively, a single WE could be used containing different electroactive labels coupled to different biorecognition elements. The obtained electrochemical signal can be resolved for the quantification of different analytes.

**Figure 16:**
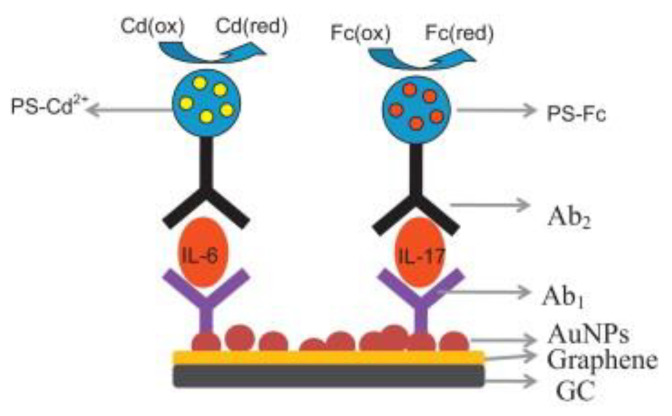
Schematic illustration of a PS-Cd^2+^-anti-IL-6 Ab, PS-Fc-anti-IL-17 Ab immunosensor for multiplexed detection of IL-6 and IL-17. Upon sandwich immunoreaction, two current peaks are generated with SWV, corresponding to the reduction in Cd^2+^ and Fc. Reprinted from [153] with permission from Elsevier.

Li et al. developed an electrochemical immunosensor for multiplexed detection of IL-6 and IL-17 using PDDA-coated polystyrene beads labeled with different metal nanoparticles. Beads conjugated to anti-IL-6 Ab_2_ were labeled with Cd^2+^, and beads conjugated to anti-IL-17 Ab_2_ were labeled with Fc (Figure 16). SWV measurements were performed using an AuNP-functionalized GCE coated with anti-IL-6 and anti-IL-17 Ab_1_. This sensor successfully resolved two current peaks for IL-6 and IL-17 at two different voltage points. The current peak was found to sharpen upon the dissolution of polystyrene with tetrahydrofuran, in comparison to simple polystyrene incubation prior to detection [153].

Pui et al. developed an FET biosensor for the continuous detection of IL-6 and TNFα secreted by a macrophage cell culture, when stimulated with lipopolysaccharide. APTES-glutaraldehyde pair was used to immobilize anti-TNFα Ab and anti-IL-6 Ab on two different silicon nanowire (SiNW) gates. The gates were connected to individual drains, but shared a common source. Changes in conductance were used to determine the antigen concentration. IL-6 and TNFα were also determined from rat serum samples stimulated with lipopolysaccharide, and the results were compared with that of ELISA [154]. Baraket et al. reported a biosensor for multiplexed IL-10 and IL-1β detection using two 4-carboxymethyl aryl diazonium-grafted Au WEs, one functionalized with anti-IL-10 Ab and the other with anti-IL-1β Ab. Cytokine concentration was detected using EIS [155].

**Figure 17:**
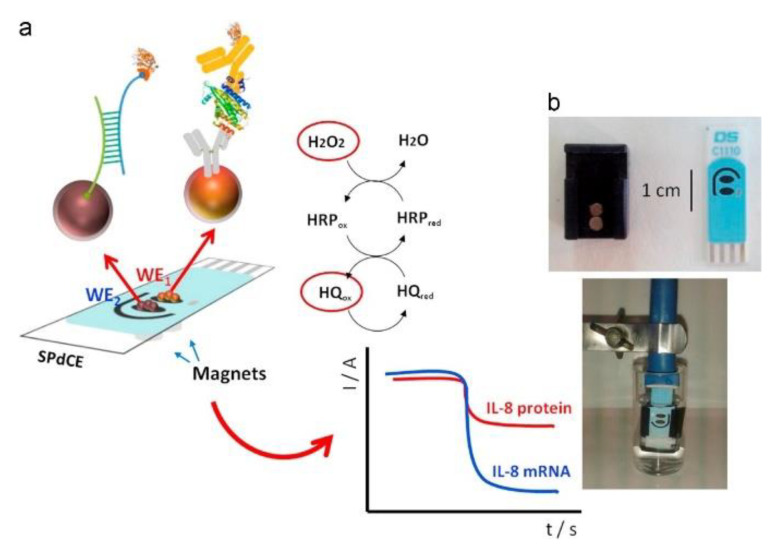
(**a**) Schematic illustration of a magnetoimmunosensor for the simultaneous determination of IL-8 mRNA and IL-8. (**b**) Photograph of the SPdCE and the magnet holding block (top), and the modified Mbs on the SPdCE assembled on the magnet holding block dipped in a solution (bottom). Reprinted from [156] with permission from Elsevier.

Torrente-Rodriguez et al. designed a magnetoimmunosensor for the quantification of IL-8 mRNA and IL-8 protein in saliva samples. Test solutions were spiked with IL-8 protein and an associated synthetic DNA oligonucleotide representing IL-8 mRNA. Anti-IL-8 Ab and cDNA (complementary to IL-8 mRNA) were immobilized on magnetic beads. A HRP (catalyst)-hydroquinone, H_2_O_2_ (substrates) system was employed for amperometric detection on a screen printed dual carbon electrode (SPdCE), coupled with a neodymium magnet for Mb separation (Figure 17). The detection limit of IL-8 protein obtained here (72.4 pg/mL) was about 23 times lower than the cutoff (600 pg/mL) set for oral cancer patients. The ability to measure the transient expression of cytokines, both mRNA and protein simultaneously, can provide meaningful insight into the progression of an immunological response [156].

A summary of design and sensor preparation process as well as sensor performance parameters are presented on Table 17 and Table 18.

**Table 17:** Biosensors for multiplexed cytokine detection: Design and sensor preparation.

Sl. No. Ref.	Transducer Components	Redox Species	Bio-Recognition Element	Blocking Agent	Preparation and Immobilization (Brief)
1. [153]	GCE/Graphene-Chitosan/AuNP/anti-IL-6 Ab_1_, anti-IL-17 Ab_1_ PS-Cd^2+^/PDDA/AuNP/anti-IL-6 Ab_2_ PS-Fc/PDDA/AuNP/anti-IL-17 Ab_2_	Cd^1+/2+^ Fe^2+/3 +^ of Ferrocene	Anti-IL-6 Ab_1_, Ab_2_ Anti-IL-17 Ab_1_, Ab_2_	BSA	PVP/C_2_H_5_OH + AIBN + St + Cd(NO_3_)_2_: synthesis of PS-Cd^2+^ PVP/C_2_H_5_OH + AIBN + St + Fc: synthesis of PS-Fc PS-Cd^2 +^ or PS-Fc + PDDA: coating PS/PDDA(+ve) + AuNP(-ve): electrostatic adsorption PS/PDDA/AuNP + Ab_2_ (anti-IL-6 for PS-Cd^2 +^ & anti-IL-17 for PS-Fc): adsorption Graphene + Chitosan + AuNP: functionalization Graphene-Chitosan/AuNP + anti-IL-6 Ab_1_, anti-IL-17 Ab_1_: adsorption; drop-casted on GCE Tetrahydrofuran (THF) added to sensor for PS dissolution, followed by evaporation
2. [154]	(1) SiNW/APTES-Glu-anti-IL-6 Ab gate (2) SiNW/APTES-Glu-anti-TNFα Ab gate	-	Anti-IL-6 Ab Anti-TNFα Ab	Ethanolamine	SiNW fabricated with top-down method SiNW (surface layer SiO_2_) + APTES: silanization SiNW/APTES(-NH_2_ gr.) + Glu(-CHO gr.): imine bond formation SiNW/APTES/Glu(-CHO gr.) + Ab(-NH_2_ gr.): imine bond formation IL-6, TNFα secreted: (i) by macrophage cell line, stimulated by bacterial endotoxin lipopolysaccharide (LPS), measured every 3 h; (ii) in rat serum stimulated by LPS
3. [155]	(1) Si/SiO_2_/Ti-Ni-Au/CMA/anti-IL-10 Ab (2) Si/SiO_2_/Ti-Ni-Au/CMA/anti-IL-1β Ab	[Fe(CN)_6_]^4−/3−^	Anti-IL-10 Ab Anti-IL-1β Ab	-	Ti, Ni, Au trilayer deposited on SiO_2_/Si by physical vapor deposition; microelectrode designed by photolithography + etching Si/SiO_2_/Ti-Ni-Au + CMA: reduction, grafting with CV Si/SiO_2_/Ti-Ni-Au/CMA + Ab: amide bond formation with EDC/NHS catalyst
4. [156]	SPdCE (1) IL-8 protein WE: Mb-Ab Bt-Ab_2_ SAv-HRP (2) IL-8 mRNA WE: Mb-SAv Bt-hcDNA SAv-HRP Bt-tDNA	Hydroquinone/benzoquinone H_2_O_2_/H_2_O	Ab, hcDNA	Ethanolamine	Mb-COOH + Ab_1_(-NH_2_ gr.): amide bond formation with EDC/NHS catalyst Bt + SAv: affinity bonding mRNA associated synthetic oligonucleotide (tDNA) biotinylated separately Mbs separated from sample with magnetic separator; immobilized on SPdCE with neodymium magnet

Common abbreviations: AuNP = Gold nanoparticles, GCE = Glassy carbon electrode, PS = Polystyrene, PDDA = Poly(diallyldimethylammonium chloride), Fc = Ferrocene, SiNW = Silicon nanowires, APTES = (3-aminopropyl)triethyoxy-silane, Glu = glutaraldehyde, CMA = 4-carboxymethyl aryl diazonium salt, SPdCE = Screen printed dual carbon electrode, Mb = magnetic bead, HRP = Horseradish peroxidase, Bt = Biotin, SAv = Streptavidin, hcDNA = hairpin, complementary DNA, tDNA = target, synthetic DNA; Ab_1_ = capture antibody, Ab_2_ = signal antibody; EDC = 1-ethyl-3-(3-dimethylaminopropyl)carbodiimide, NHS = N-hydroxysuccinimide, PVP = Polyvinylpyrrolidone, AIBN = 2,2’-azobisisobutyronitrile, St = Styrene.

**Table 18:** Biosensors for multiplexed cytokine detection: Specifications and performance characteristics.

Sl. No. Ref.	Detection Technique	Limit of Detection	Range of Detection	Interfering Species Tested	Incubation Time	Sample Type	Reproducibility	Stability	Repeatability
1. [153]	SWV	IL-6: 0.5 pg/mL IL-17: 1 pg/mL	IL-6: 1–1000 pg/mL IL-17: 2–1000 pg/mL	PSA, hIgG, TNFα	+IL-6, IL-17/1 h +PS-Cd^2+^/Ab_2_, PS-Fc-Ab_2_/1 h	IL-6, IL-17 in buffer Human serum sample	(5 assays, at [IL-6] = 10 pg/mL, [IL-17] = 10 pg/mL) RSD ≤ 4.8%	-	-
2. [154]	FET based detection	IL-6: 100 fg/mL TNFα: 100 fg/mL	-	Leptin, Resistin	(real-time)	TNFα, IL-6 in: PBS DMEM/FBS culture media Rat serum sample	-	-	-
3. [155]	EIS	IL-10: 0.3 pg/mL IL-1β: 0.7 pg/mL	IL-10: 1–15 pg/mL IL-1β: 1–15 pg/mL	IL-6	+IL-10 or IL-1β/30 min	IL-10 or IL-1β in buffer	-	-	-
4. [156]	CA	IL-8 mRNA: 0.21 nM IL-8 protein: 72.4 pg/mL (undiluted saliva)	-	(1) IL-8 mRNA WE: Non-complementary DNA, single base-mismatched DNA (2) IL-8 protein WE: IL-6, lysozyme, LPO	(1) Mb-SAv+ Bt-hcDNA/60 min + IL-8, Bt-tDNA/30 min +SAv-HRP/15 min (2) Mb-Ab_1_+ IL-8, Bt-tDNA/30 min +Bt-Ab_2_/30 min +SAv-HRP/45 min	IL-8, Bt-tDNA in: PBS-Tween20 Spiked saliva Undiluted saliva samples	(5 assays, at [IL-8 mRNA] = 2.5 nM, [IL-8 protein] = 600 pg/mL, PBST buffer) RSD_IL-8 mRNA_ = 7.7% RSD_IL-8 protein_ = 8.3%	Signal within control limits: Within 11 days for IL-8 mRNA; Within 30 days for IL-8 protein	(disposable)

Common abbreviations: PBS = Phosphate-buffered saline, BSA = Bovine Serum Albumin, hIg = Human immunoglobulin protein, PSA = Prostate specific antigen, DMEM = Dulbecco’s Modified Eagle Medium, FBS = Fetal bovine serum, LPO = Lactoperoxidase.

## 3. Conclusion and Future Perspectives

Cytokines are an important class of molecules that hold tremendous potential for the prognosis and diagnosis of a broad range of conditions and illnesses, including cancer, autoimmune diseases and neurodegenerative diseases. Within the past decade, a broad range of electrochemical sensors have been reported for the quantification of various cytokines, including IL-6, TNFα and IFNγ, which hold promise as disease biomarkers. A wide variety of nanomaterials, biomolecules and sensing schemes have been employed to enhance the sensor performance, such as the detection sensitivity, specificity and reproducibly. For instance, enzyme-labeled immunosensors are some of the earliest electrochemical biosensors that were designed for detecting cytokines. Replacing enzymes with non-enzymatic labels, such as redox labels and heavy metal nanoparticles, improved the stability of the sensor to a favorable extent. Magnetoimmunosensors, on the other hand, provided a separate site for the immunoreaction to take place, away from the electrode. This led to further enhancement of stability and also ensured hassle-free regeneration and reusability of the magnetoimmunosensors. Label-free detection offered by impedimetric and FET-biosensors are by far the most simple and cost-effective sensing technologies available. It is also noteworthy that aptasensors offer better shelf life and flexibility of design than immunosensors, due to the stability and versatility of synthetically manufactured and in vitro selected aptamers. Furthermore, FET-biosensors and screen-printed biosensors are compact and miniaturized platforms, and present the benefits of cost-effective production, portability and ease of handling. Based on these approaches, many of which have been utilized in works discussed in this review, we see that much progress has been made toward the development of simple, low-cost electrochemical biosensors for sensitive cytokine measurements.

However, there are still several challenges that need to be overcome in order for this technology to be acceptable for routine clinical testing. To the best of our knowledge, no electrochemical biosensors have been made commercially available, so far, for the detection of cytokines. The stability (shelf life) of most electrochemical sensors ranges from only a few days to weeks. In comparison, the shelf life of many commercial ELISA tests ranges from several months up to one year when stored at 4 °C/−20 °C. The reason that electrochemical sensors exhibit such lower stability is because the transducing electrodes are typically pre-assembled with various SAMs, nanomaterials and biomolecules, and the integrity of these assemblies can degrade over time. For example, most of the biosensors discussed in this review were tested immediately after being fabricated, and some reported a significant loss in the detection signal intensity when stored for a few weeks. In contrast, most ELISA tests require biomolecular assembly prior to each measurement. Therefore, future efforts should focus on extending the shelf life of electrochemical biosensors through the use of shelf-stable sensor components and biomolecular stabilizing agents. Another challenge is obtaining high-sensitivity cytokine measurements in raw biofluid samples, such as blood, saliva and urine. Many of the cytokine biosensors that have been reported were tested using buffer samples or diluted matrices due to the high content of interfering species present in raw biofluids. Additional research is necessary to enhance the detection sensitivity and specificity in order to detect cytokines at clinically relevant levels in raw biofluids.

As discussed earlier, cytokines are expressed transiently, and their pathways intersect at several points. Therefore, cytokine profiling can offer valuable information about the type, severity and route of progression of various pathophysiological conditions, which can also be used to assess the efficacy of immunotherapeutic treatments. Cytokine profiling can be especially helpful in the assessment of disease conditions accompanied by the overactivation of the immune system, as a primary source of physiological damage. In particular, the cases of COVID-19 infections exhibit such phenomenon, marked by cytokine storms, acute lung inflammation and, in extreme cases, multi-organ dysfunction and failure. With this in mind, the development of electrochemical biosensors for multiplexed detection of cytokines is a promising research direction. Furthermore, the utility of multiplexed biosensors can be further extended to studying the effect of cytokines as therapeutic drugs in cell lines. This brings us to another promising application of electrochemical sensors: monitoring cell lines. Immune cell lines are routinely cultured and maintained in cancer research, immunology and infectious disease laboratories. These fields could greatly benefit from the development of electrochemical biosensors that can be integrated with cell cultures through microfluidic platforms. A couple of works discussed in this review have already attempted this. An integrated microfluidic sensing platform will not only offer ease of handling, but also reduce the chances of contamination, and enable continuous cytokine screening. However, there are some practical aspects, such as limited stability/viability of cell lines in such integrated platforms, electrode biofouling and so on, that have yet to be addressed. Lastly, accurate cytokine detection in noninvasive bodily fluids, such as saliva, sweat or urine, can be useful, particularly for infants and individuals with poor blood clotting (hemophilia).

## Data Availability

Data sharing not applicable.
